# Impact of endocrine disruptors on peripheral blood mononuclear cells in vitro: role of gender

**DOI:** 10.1007/s00204-023-03592-3

**Published:** 2023-09-07

**Authors:** Ambra Maddalon, Luigi Cari, Martina Iulini, Mahdieh Naghavi Alhosseini, Valentina Galbiati, Marina Marinovich, Giuseppe Nocentini, Emanuela Corsini

**Affiliations:** 1 Laboratory of Toxicology, Department of Pharmacological and Biomolecular Sciences, Rodolfo Paoletti’, Università Degli Studi Di Milano, Via Balzaretti 9, 20133 Milan, Italy; 2 Department of Medicine and Surgery, Section of Pharmacology, Università Degli Studi Di Perugia, Building D, Severi Square 1, 06129 Perugia, Italy

**Keywords:** Endocrine active compound, Immunomodulation, Immunotoxicity, Peripheral blood mononuclear cells, T cells, NK cells, Sex effects

## Abstract

Humans can be exposed to endocrine disruptors (EDs) in numerous ways. EDs can interfere with endogenous hormones at different levels, resulting in numerous adverse human health outcomes, including immunotoxicity. In this regard, this study aimed to investigate in vitro the possible effects of EDs on immune cells and possible gender differences. Peripheral blood mononuclear cells from healthy humans, both males and females, were exposed to 6 different EDs, namely atrazine (herbicide), cypermethrin (insecticide), diethyl phthalate (plasticizer), 17α-ethynylestradiol (contraceptive drug), perfluorooctanesulfonic acid (persistent organic pollutant), and vinclozolin (fungicide). We evaluated the effect of EDs on RACK1 (receptor for activated C kinase 1) expression, considering it as a bridge between the endocrine and the immune system, and putatively used as screening tool of immunotoxic effects of EDs. The exposure to EDs resulted at different extent in alteration in RACK1 expression, pro-inflammatory activity, natural killer lytic ability, and lymphocyte differentiation, with sex-related differences. In particular, diethyl phthalate and perfluorooctanesulfonic acid resulted the most active EDs tested, with gender differences in terms of effects and magnitude. The results from our study evidenced the ability of EDs to directly affect immune cells.

**Supplementary Information:**

The online version contains supplementary material available at 10.1007/s00204-023-03592-3.

## Introduction

According to the definition by World Health Organization (WHO), ‘an endocrine disruptor (ED) is an exogenous substance or mixture that alters function(s) of the endocrine system and consequently causes adverse health effects in an intact organism, or its progeny, or (sub)populations (WHO 2002)’. Compounds with possible endocrine activities can be found in consumer products, food-contact materials, plasticizers, pharmaceuticals, and pesticides (Kuo et al. 2012). Therefore, humans are daily exposed to EDs (Yilmaz et al. 2020). Hormones modulate the homeostasis of many systems, including the immune system. The link between the endocrine and the immune system is well established, and it is known that the immune function can be targeted by EDs (Greives et al. 2017). Both in vivo and in vitro evidence highlighted the interaction between EDs and the immune system with multiple targets and processes (Chalubinski and Kowalski 2006; Rogers et al. 2013; Bansal et al. 2018; Nowak et al. 2019; Masi et al. 2021; Patisaul 2021; D’Amico et al. 2022a). In this study, we selected six different EDs, covering a range of different uses: diethyl phthalate (DEP), 17α-ethynylestradiol (EE), perfluorooctanesulfonic acid (PFOS), atrazine (ATR), cypermethrin (CYP), and vinclozolin (VIN). DEP is a phthalate ester widely used in industry as plasticizer, fixative, and solvent in cosmetics and packaging materials (Kamrin and Mayor 1991; Api 2001). EE is a derivative of estradiol used in contraceptive pills. PFOS is a man-made fluorosurfactant and global pollutant (Liang et al. 2022; EU 2019). ATR is an herbicide, which, although was banned in Europe, still represents a contamination issue due to its presence in waters and soils (Bhatti et al. 2022). CYP is a pyrethroid insecticide and VIN is a fungicide (Hrelia et al. 1996; Behnami et al. 2021; Kanyika-Mbewe et al. 2020). High levels of these EDs were found in drinking and surface waters, indicating their high exposure to humans (SCHER 2011; Net et al. 2015; Domingo and Nadal 2019; Tang et al. 2021; Li et al. 2022a).

Adverse effects on the immune system were observed with several EDs, including bisphenols, phthalates, and several pesticides (Patisaul 2021; Schjenken et al. 2021). These substances can interfere with the development and function of the immune system, acting on both innate and adaptive responses (Ahmed 2000; Chalubinski and Kowalski 2006; Rogers et al. 2013; Bansal et al. 2018; Buoso et al. 2021; Galbiati et al. 2021; Maddalon et al. 2022). Within the selected EDs, DEP is a phthalate compound, and in general, thus, family is considered to have endocrine disrupting properties (Hlisníková et al. 2020). The ability of DEP to mimic estrogen and activate the estrogen receptor has been assessed (Fiocchetti et al. 2021). Regarding the effects on the immune system, the few literature data available suggest a possible effect on it, like the induction of immune-related genes (Xu et al. 2013), but being a phthalate, a similar action is suggested (Hansen et al. 2015). EE, being a drug used for birth control, has effects on the endocrine system, mainly regarding estrogen pathways. Its adverse effects on the immune system were observed in animal models, but studies in humans are sparse (Klinger et al. 2000; Cabas et al. 2012; Massart et al. 2014; Kernen et al. 2022). PFOS has been linked to both thyroid and reproductive dysfunctions (Coperchini et al. 2017; Tarapore et al. 2021), being able to affect hormone receptors and genes related to endocrine function (Du et al. 2013). Furthermore, its adverse action on immunity has been extensively investigated (Qazi et al. 2010; Guo et al. 2019; Torres et al. 2021; Liang et al. 2022), indicating the reduced antibody response following vaccination as the critical effects (EFSA Panel on Contaminants in the Food Chain 2018). ATR, which was associated with reproductive dysfunctions (Chevrier et al. 2011; Hayes et al. 2011; Goodman et al. 2014; Namulanda et al. 2017; Almberg et al. 2018; Griffiths et al. 2022; Owagboriaye et al. 2022;), due to its ability to affect androgens and estrogen levels (Trentacoste et al. 2001; Eldridge et al. 2008), is able also to affect immune functionality, mainly inducing immunosuppression and acting on T cells (Filipov et al. 2005; Pinchuk et al. 2007; Rowe et al. 2008; Zhao et al. 2013; Lee et al. 2016; Chang et al. 2021; Galbiati et al. 2021). CYP is considered able to alter immune functionality in rats (Liu et al. 2006) and exert myelotoxicity in human cells (Mandarapu and Mrakhya 2015). The endocrine effects of CYP are debated, and several evidences indicate its ability to interfere with the endocrine system (Jin et al. 2011; Singh et al. 2020; Irani et al. 2022; Li et al. 2022b), but recently it has been classified as unlikely to cause endocrine disruption (EC 2019). Finally, VIN effects on the endocrine system have been reported, evidencing altered male reproduction as the main effect, inducing a lower sperm quality and number, epididymal morpholohical changes, and prostate abnormalities (Anway and Skinner 2008; Paoloni-Giacobino 2014; Feijó et al. 2021). Regarding the action on the immune system, only few information are available, namely its ability to interact with NF-κB and with lymphocyte activity, increasing T and B cells percentage, while decreasing NK cells (White et al. 2004; D’Amico et al. 2022b).

The EDs have been selected based on their different endocrine targets (i.e., hormone receptors, enzymes, hormone synthesis). Indeed, EE is able to interfere with the estrogen pathway, DEP can act on estrogen and glucocorticoid pathway, while PFOS can impair the estrogen, glucocorticoid and thyroid signaling (Masi et al. 2022). Furthermore, ATR is able to interact mainly on the androgen pathway, but it can also interfere with estrogen and aromatase activity, whereas CYP acts indirectly on the androgen receptors, and VIN can act both on the androgen and estrogen pathways (Maddalon et al. 2022). The selected EDs were analyzed to assess their ability to interfere with a protein that represents a bridge between the endocrine and the immune system. Recently, we demonstrated that these EDs were able to modulate monocytes’ activation in vitro, through the modulation of RACK1 (receptor for activated C kinase 1) (Maddalon et al. 2022; Masi et al. 2022). This latter was identified as a target of EDs in the immune system and as a possible link between these two systems, since it is involved in the activation of innate immunity and represents a relevant target of endocrine action (Buoso et al. 2017; 2020). RACK1 expression, being under hormonal control, could be able to integrate the signals of different EDs and therefore influencing the immune response. This protein could serve as screening tool to evaluate the immunotoxic profile of EDs.

The purpose of this study was to evaluate the in vitro effects of the selected EDs on several immunological endpoints using primary cultures of human peripheral blood mononuclear cells (PBMC). Their ability to modulate RACK1 expression, to interfere with natural killer (NK) cell activity, and lymphocyte differentiation, focusing on CD4^+^ and CD8^+^ cells, was investigated.

## Materials and methods

### Tested chemicals

The selected EDs are listed in Table 1, together with their acronym, CAS number, and the tested concentration.

**Table 1:** ED tested: name, acronym, CAS number, and concentration used

Name	Acronym	CAS N°	Concentration (μM)
Diethyl phthalate	DEP	84-66-2	1
17α-ethynylestradiol	EE	57-63-6	0.001
Perfluorooctanesulfonic acid	PFOS	1763-23-1	0.2
Atrazine	ATR	1912-24-9	1
Cypermethrin	CYP	52315-07-8	1
Vinclozolin	VIN	50471-44-8	0.1

All the substances were purchased from Sigma-Aldrich (St. Louis, Missouri, US) at the highest purity available. They were dissolved in dimethyl sulfoxide (DMSO; CAS # 67-68-5, purity ≥ 99.5%) at 10 mM stocks that were stored at − 20 ℃. Working concentrations were then obtained diluting stock solutions for each treatment. The final DMSO concentration in culture medium was ≤ 0.2%, and it was used as solvent control. Concentrations were selected based on previous studies conducted on THP-1 cell line (Maddalon et al. 2022; Masi et al. 2022), as the lowest concentration active on at least one immune parameter. Preliminary experiments were conducted to ensure that the concentrations used were not cytotoxic, as assessed by propidium iodide (PI) staining and flow cytometric analysis (data not shown).

### PBMC treatment with EDs

PBMCs were obtained by Ficoll gradient centrifugation from buffy coats from anonymous healthy blood donors of both sexes, purchased from the Niguarda Hospital in Milan (Italy). Following centrifugation, PBMC layers were removed, and after washing with Dulbecco’s Phosphate-Buffered Saline (PBS), isolated cells were diluted to 10^6^ cells/mL or 5 × 10^6^ cells/mL, based on the treatment, in RPMI-1640 without phenol red, containing 2 mM L-glutamine, 0.1 mg/mL streptomycin, 100 IU/mL penicillin, 10 µg/mL gentamycin, 50 µM 2-mercaptoethanol, supplemented with 5% heat-inactivated dialyzed fetal bovine serum (culture medium) and cultured at 37 ℃ in a 5% CO_2_ incubator.

For the evaluation of RACK1 expression, PBMCs (10^6^ cells/mL) were exposed to the different EDs or DMSO (vehicle control) for 24 h at 37 °C in a 5% CO_2_ incubator. RACK1 protein expression was evaluated by Western blot analysis and normalized to β-tubulin expression.

To evaluate the expression of CD86 and CD54, and the release of IL-8 and TNF-α, PBMCs (10^6^ cells/mL), following 24 h of exposure to EDs or DMSO, were stimulated with lipopolysaccharide (LPS) from *Escherichia coli* serotype 0127:B8 (Sigma-Aldrich) at the final concentration of 100 ng/mL for further 24 h at 37 ℃ in a 5% CO_2_ incubator.

To evaluate NK-cell lytic activity, PBMCs (5 × 10^6^ cells/mL) were exposed to the different EDs or DMSO for 24 h. As target cells, K562 cells (AddexBio, US) stained with CellTrace™ CFSE (Invitrogen, Waltham, Massachusetts, US) were used. Briefly, 500 µL of K562 at the concentration of 10^6^ cells/mL were centrifuged, the CellTrace™ CFSE was added to the cell pellet (1:1000 dilution) and incubated for 15 min at 37 ℃ protected from light. After the incubation time, the reaction was stopped by adding culture medium containing 5% heat-inactivated dialyzed fetal bovine serum. CellTrace™ CFSE-stained K562 cells were then kept to the concentration of 10^5^ cells/mL and co-cultured together with EDs/DMSO-exposed PBMCs. Three different ratios of effector (PBMC) and target (K562) cells were used: 50:1, 25:1, 12.5;1, maintaining fixed concentration of K562 cells. The cellular concentrations are reported in Table 2.

**Table 2:** Ratio between effector and target cells to assess NK cell’s lytic activity

Treatment ratio (effector:target)	Effector (PBMC)	Target (K562)
50:1	5 × 10^6^ cells/mL	10^5^ cells/mL
25:1	2.5 × 10^6^ cells/mL	10^5^ cells/mL
12.5:1	12.5 × 10^6^ cells/mL	10^5^ cells/mL

The cells are then co-cultured for 4 h at 37 ℃ in a 5% CO_2_ incubator.

For the assessment of T-cell differentiation, 25 µL of Dynabeads™ Human T-Activator CD3/CD28 for T-Cell Expansion and Activation (ThermoFisher, Waltham, Massachusetts, US) was added to 10^6^ PBMCs. Cells were then exposed to the EDs or DMSO and incubated for 4 days at 37 °C in a 5% CO_2_ incubator.

### Immunoblot analysis of RACK1 expression

After 24 h of treatment, cells were harvested, washed, and lysed in homogenization buffer (50 mM Tris–HCl pH 7.5, 150 mM NaCl, 5 mM EDTA, 0.5% Triton X-100 and protease inhibitor). Protein content was assessed using the Bradford method. Cell lysates were mixed with sample buffer (125 mM Tris–HCl pH 6, 8.4% sodium dodecyl sulfate, 20% glycerol, 6% β-mercaptoethanol, 0.1% bromophenol) and denatured at 95 ℃ for 10 min. 10 µg of extracted proteins were electrophoresed into 10% SDS-PAGE under reducing conditions and then transferred to PVDF membranes. The membranes were blocked with 1X TBS, 0.1% Tween-20, and BSA (5% w/v), and the expression of RACK1 and β-tubulin assessed following over-night incubation of the relative antibodies (dilution 1:1000) and following 1-h incubation of secondary IgG peroxidase-conjugated antibodies (dilution 1:15,000). Anti-human-RACK1 mouse antibody was purchased from Santa Cruz Biotechnology (B-3 clone; Dallas, Texas, US), anti-human-β-tubulin rabbit antibody was purchased from Novus Bio (R&D Systems, Minneapolis, Minnesota, US), goat anti-mouse IgG was purchased from Sigma-Aldrich, and goat anti-rabbit IgG was purchased from Bio-Rad. All the antibodies were diluted in 1X TBS, 0.1% Tween-20, and BSA 5% w/v. The band visualization was performed using Clarity western ECL blotting substrates (Bio-Rad, Hercules, California, US). Blot images were acquired with Image Lab Software version 4.0 (Bio-Rad) using the Molecular Imager Gel Doc XR (Bio-Rad) and quantified normalizing on β-tubulin expression levels. The stimulation index (SI) was calculated on DMSO-treated PBMCs (vehicle control) set at 100.

### Flow cytometric analysis of CD86 and CD54 expression

After 48 h of treatment, PBMCs were centrifuged, and the supernatants were stored at -20° C for the assessment of cytokine release. Cell pellets were washed with PBS, suspended in 200 µl of PBS, and stained at 4° C in the dark for 30 min with specific PE-conjugated antibody against human CD54 or FITC-conjugated antibody against human CD86 or with isotype control antibodies, following supplier’s instructions. All the antibodies were purchased from BD Biosciences (Franklin Lakes, New Jersey, US). After incubation, cells were centrifuged and suspended in 500 µL of PBS. The % of positive cells was analyzed using Novocyte 3000 flow cytometer (Acea Bioscience Inc., Agilent Technologies, Santa Clara, California, US) and data were quantified using Novocyte software (Acea Bioscience Inc.). 10′000 viable cells were analyzed for % of positivity to the respective marker. The % of isotype control was subtracted from the % of CD86/CD54 stained cells. Changes in CD86/CD54 expression are reported as SI calculated on DMSO-treated PBMC (vehicle control) set at 1. The gating strategy is reported in Supplementary Fig. 1 and representative dot plots are reported in Supplementary Fig. 2.

### Cytokine production

From the same treatments in which surface markers expression was assessed, the cell-free supernatants were kept at – 20 ℃ for cytokine evaluation through commercially available ELISA kits. The ELISA kits to assess the release of IL-8 and TNF-α were purchased from ImmunoTools (Friesoythe, Germany) and R&D Systems, respectively. The limits of detection were 8 pg/mL for IL-8 and 7.8 pg/mL for TNF-α, respectively. Changes in IL-8/TNF-α release are reported as SI calculated on DMSO-treated PBMC (vehicle control) which was set at 1.

### Assessment of the lytic activity of NK cells

After the treatment with EDs and the co-culture with CellTrace™ CFSE-stained K562 cells, the plate content was transferred to flow cytometrical tubes and PI (5 nM) was added to each tube. The % of cells positive to PI within CFSE-stained K562 cells was acquired using Novocyte 3000 flow cytometer. 1′000 CFSE-positive cells were analyzed for % of positivity to PI, indicative of dead K562 cells. The gating strategy is reported in Supplementary Fig. 3 and representative dot plots are reported in Supplementary Fig. 4.

### Flow cytometric analysis of T cells differentiation

After 4 days of treatment, the Protein Transport Inhibitor Cocktail (Invitrogen) was added to each well, to stop cytokine secretion (1 µL every 500 µL of cell culture), for 5 h at 37 ℃ in a 5% CO_2_ incubator. Following incubation, cells were harvested and washed, magnetic beads were removed using DynaMag 15 (Invitrogen) and washed again. The staining for surface markers was then performed, according to Table 3, for 30 min at room temperature avoiding the light.

**Table 3:** Antibodies used to stain PBMC, their dilution, the channel used to acquire them at the flow cytometer, and the supplier

Antigen	Clone	Dilution	Channel	Supplier
Surface antibodies				
GITR	DT5D3	1:200	BL2	Miltenyi
CD8	OKT8	1:200	VL2	ThermoFisher
CD4	OKT4	1:200	VL3	ThermoFisher
CD25	BC96	1:200	VL4	ThermoFisher
Intracellular antibodies				
IL-4	8D4-8	1:200	VL1	ThermoFisher
IL-9	MH9D1	1:200	BL3	ThermoFisher
IL-10	JES3-9D7	1:200	BL1	ThermoFisher
IL-17A	eBio64DEC17	1:200	RL3	ThermoFisher
IL-22	IL22JOP	1:200	RL1	ThermoFisher
IFN-γ	4S.B3	1:200	RL2	ThermoFisher
FoxP3	236A/E7	1:200	BL4	ThermoFisher

After incubation, cells were washed and fixed using the fixation reagent for 45 min on ice avoiding the light. After washing, cells were permeabilized using permeabilization reagent for 5 min on ice avoiding the light and washed again, following supplier’s instruction (eBioscience™ Foxp3 / Transcription Factor Staining Buffer Set—Invitrogen). Then, cells were stained for intracellular proteins, according to Table 3, for 3 h and 30 min at 4 °C avoiding the light. After that, 1 ml of PBS with 1% of fetal bovine serum was added, the cells were filtered using pre-separation filters (70 μM—Miltenyi Biotec, US) and the samples acquired using the Attune NxT flow cytometer (ThermoFisher). Data were further analyzed through FlowJo V.10.8.1 (BD Biosciences). The % of positive cells to the different markers was retrieved. Data are presented as Log2 values calculated on DMSO-treated PBMC (vehicle control) which is set at 0. The gating strategy is reported in Supplementary Fig. 5.

### t-SNE analysis of T helper cell subpopulations

Following conventional analysis, through FlowJo, to deeply investigate T-cell subpopulations, the t-distributed stochastic neighbor embedding (t-SNE) algorithm was applied. All the.FCS files were merged in two concatenated files as follows: a) DMSO, DEP, EE, and PFOS from 6 donors (3 males and 3 females) due to their high presence in the environment and high exposure levels to humans to and b) DMSO, ATR, CYP, and VIN from 6 donors due to their belonging to the pesticide class. Samples were down-sampled to 10′000 cells, and after the gating of the single populations within CD4^+^ cells, as performed for the conventional analysis (Supplementary Fig. 5), the t-SNE was run. Briefly, a Barnes-Hut t-SNE method, with a perplexity of 50 and 3000 iterations was chosen. The results were visualized in 2D t-SNE maps. The single treatment conditions were successively gated through the sample ID. Clusters of cells based on the expression level of the different analyzed markers were manually created on merged data, and the % of gated cells in each cluster was further analyzed for the single treatment conditions. Within CD4^+^ cells, it was possible to recognize different subpopulations for IFN-γ, IL-4, IL-9, FoxP3, and GITR. For IL-10, IL-17A, IL-22, and CD25 it was not possible to define distinct subpopulations. Only the subpopulations common to all the 6 donors were further evaluated, and the % of cells present in the clusters was expressed as a Log2 value calculated on DMSO-treated PBMC, which is set at 0. The t-SNE density plots are reported in Supplementary Figs. 6 and 7.

### Statistical analysis

Statistical analysis was performed using Prism version 9.4.0 (GraphPad Software, San Diego, California, US). Data were reported as mean ± standard error (SEM) or as median of 3 (only for T cells differentiation) or 5 male and female donors, as reported in figure legends. To calculate differences between the treatment, *t* test was applied, after the assessment of the normal distribution of the data through the Shapiro–Wilk test. Differences were considered statistically significant at *p* ≤ 0.05.

## Results

### EDs modify RACK1 expression in human PBMC

First, to confirm previous results obtained in THP-1 cells (Maddalon et al. 2022; Masi et al. 2022), the ability of EDs to interfere with RACK1 expression was evaluated, being RACK1 a bridge between the immune and the endocrine systems, as previously explained.

**Fig. 1:**
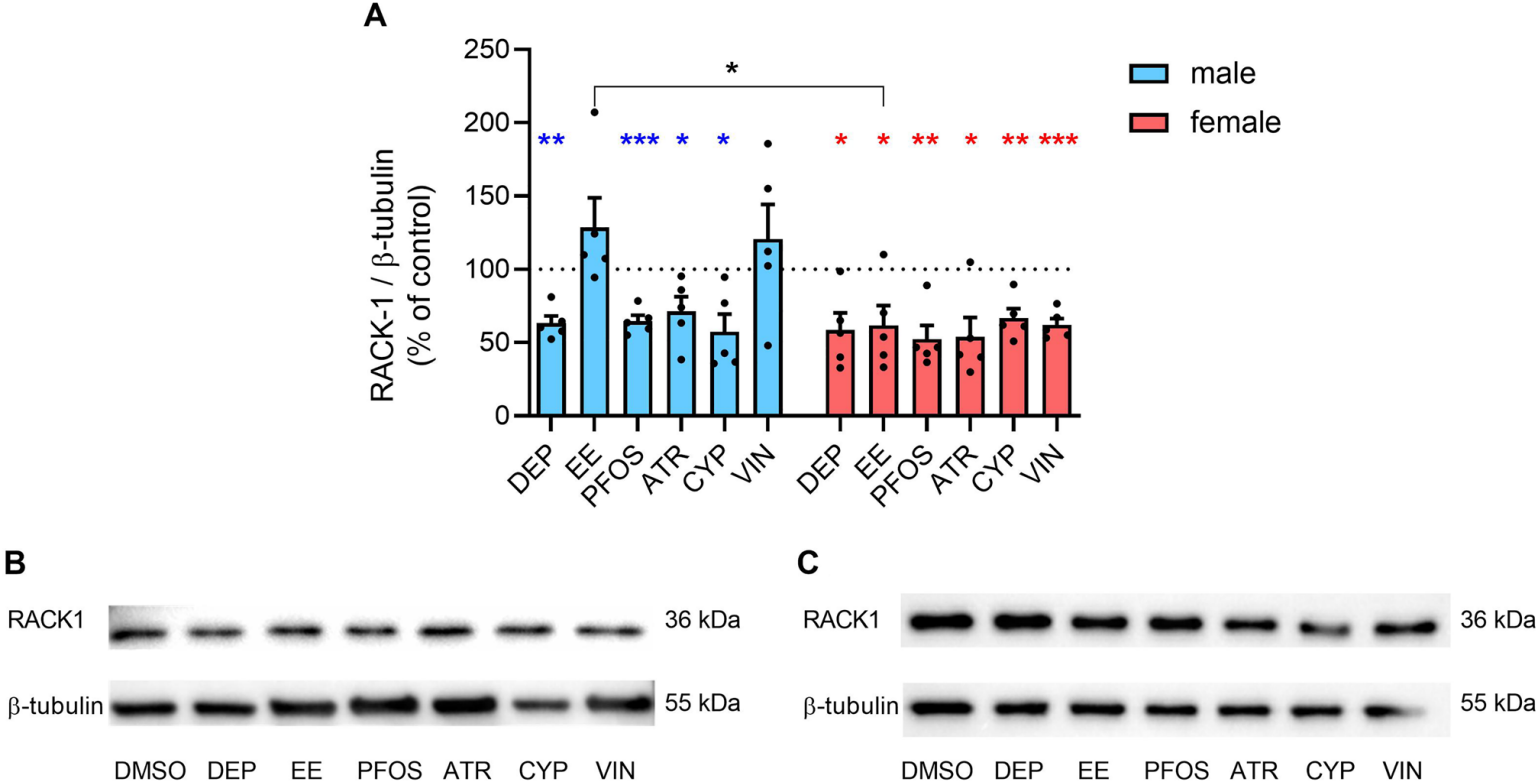
Effects of EDs on RACK1 expression. Male and female PBMC were exposed to the different EDs or DMSO (vehicle control) for 24 h. RACK1 protein level was evaluated by Western blot analysis and normalized to β-tubulin expression (**A**). Data are referred to each sample DMSO-treated PBMC (vehicle control), which is set at 100 (dotted line). Results are expressed as mean ± SEM of 5 male donors (light blue) and 5 female donors (pink). Each dot represents the expression of the single donor. Statistical analysis was performed following unpaired *t* test with Welch’s correction, with *p ≤ 0.05, **p ≤ 0.01, ***p ≤ 0.001 *vs* DMSO. Differences between males and females were assessed through unpaired *t* test, with *p ≤ 0.05 between males and females for EE exposure. **B** and **C** Representative Western blots for RACK1 and β-tubulin expression induced by EDs exposure in a representative male (**B**) and female (**C**) donor (color figure online)

Short-term exposure to different EDs resulted in changes in the expression of RACK1 in human PBMC, from both male and female healthy donors (Fig. 1). In particular, DEP, PFOS, ATR, and CYP exposure was able to induce a statistically significant reduction of RACK1 expression in PBMCs from both males and females. Whereas EE and VIN were able to down-regulate RACK1 expression in females only. Furthermore, a sex bias in the response to EE exposure was found, highlighting a possible dimorphism in its action. While in female donors all the EDs induced a reduction in RACK1 activation, and possibly a reduced immune activation, EE and VIN had no statistically significant effect in male donors at the concentration tested. The effects obtained in primary PBMCs are in line with previous results obtained in THP-1 cells. THP-1 is a human monocytic cell line derived from acute monocytic leukemia of a male patient, and results obtained with male donors are closer to the one observed in THP-1 cells. In particular, DEP, PFOS, ATR, and CYP reduced RACK1 expression in both models, whereas an increase with EE and no effect with VIN were obtained in THP-1 cells (Maddalon et al. 2022; Masi et al. 2022). The modulation of RACK1 induced by the selected EDs is also in line with the previously performed molecular docking analysis. Indeed, DEP and PFOS revealed to activate glucocorticoid receptor, that in turn reduced RACK1 expression (Masi et al. 2022). Also ATR and CYP were able to decrease RACK1 expression, but the mechanism is linked to an anti-androgenic activity: ATR is able to competitively antagonize androgen receptor, whereas CYP acts in an indirect way, reducing androgen receptor expression and IL-6 release (Maddalon et al. 2022). Also VIN is an anti-androgenic compound, but it can also activate GPER (G-protein-coupled estrogen receptor) that in turn is able to activate androgen receptor (Maddalon et al. 2022). This dual mechanism could also explain the different effect observed in male and female PBMCs. Finally, EE is characterized by an estrogenic activity, linked to the action on both GPER and androgen receptor (Masi et al. 2022). Therefore, an increase RACK1 expression would be expected, as in the case of male donors, although not statistically significant. The involvement of GPER in the activity of EE and VIN could be an hypothesis of the different gender effect.

### Effects of EDs on RACK1-related pro-inflammatory markers and cytokines production

**Fig. 2:**
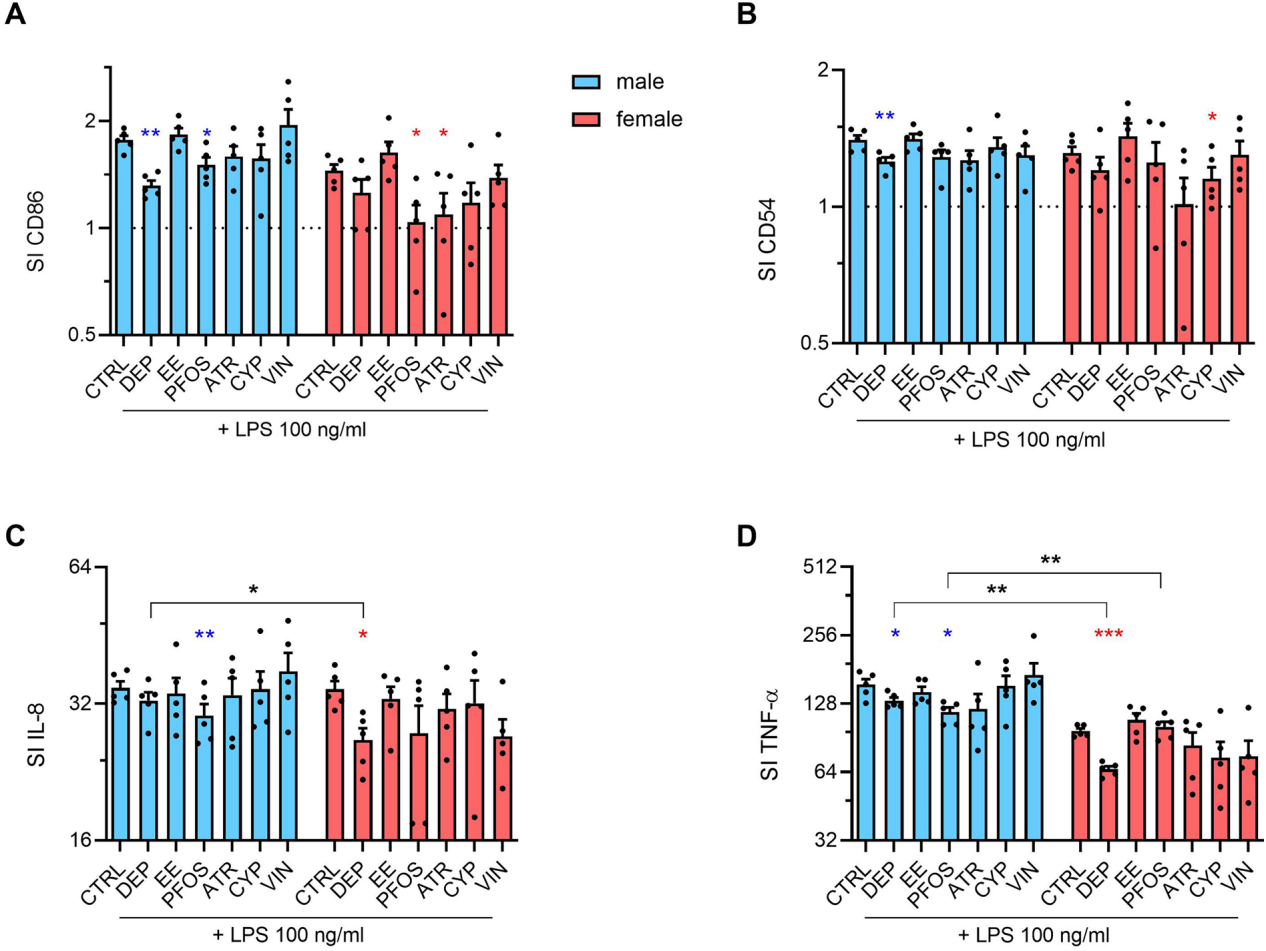
Effects of EDs on RACK1-related pro-inflammatory cytokines and activation markers. Male and female PBMC were exposed to the different EDs or DMSO (vehicle control) for 24 h and then to LPS 100 ng/ml for further 24 h. CD86 (**A**) and CD54 (**B**) expression were evaluated by flow cytometric analysis, whereas IL-8 (**C**) and TNF-α (**D**) release was assessed by ELISA. Results are expressed as SI calculated on DMSO-treated LPS-unstimulated PBMC set at 1 (dotted line) of mean ± SEM of 5 male donors (light blue) and 5 female donors (pink). Each dot represents the expression of the single donor. Note that a semi-log scale was used, to better show the results. Statistical analysis was performed following paired *t* test, with *p ≤ 0.05, **p ≤ 0.01 *vs* DMSO-treated LPS-stimulated. Differences between males and females were assessed through unpaired *t* test, with *p ≤ 0.05, **p ≤ 0.01 (color figure online)

Following the assessment of their ability to modulate RACK1 expression, the expression of CD86 and CD54 and the release of IL-8 and TNF-α were evaluated following LPS stimulation (Fig. 2). CD86 and CD54 are two surface proteins important in the process of T-cell activation, and their increase upon LPS stimulation in PBMC was observed (Fig. 2A, B).

EDs were able to modulate CD86 and CD54 expression in PBMC, with gender differences observed. The exposure to DEP was able to reduce LPS-induced CD86 and CD54 expression in male donors (Fig. 2A, B). PFOS decreased CD86 expression in both sexes (Fig. 2A). ATR and CYP exposure reduced, respectively, CD86 and CD54 expression, only in female donors (Fig. 2A, B). Similarly, the release of the pro-inflammatory cytokines IL-8 and TNF-α was increased by LPS treatment and it was modified by EDs pre-incubation (Fig. 2C, D). In detail, PFOS was able to reduce both IL-8 and TNF-α release in male donors only, and in case of TNF-α, a statistically significant difference between males and females was observed (Fig. 2C, D). Sex differences were obtained also following DEP stimulation, inducing a decrease in IL-8 release in females only, and of TNF-α in both sexes, but with a higher susceptibility of female donors (Fig. 2C, D).

### Effect of EDs on NK cells' lytic activity

**Fig. 3:**
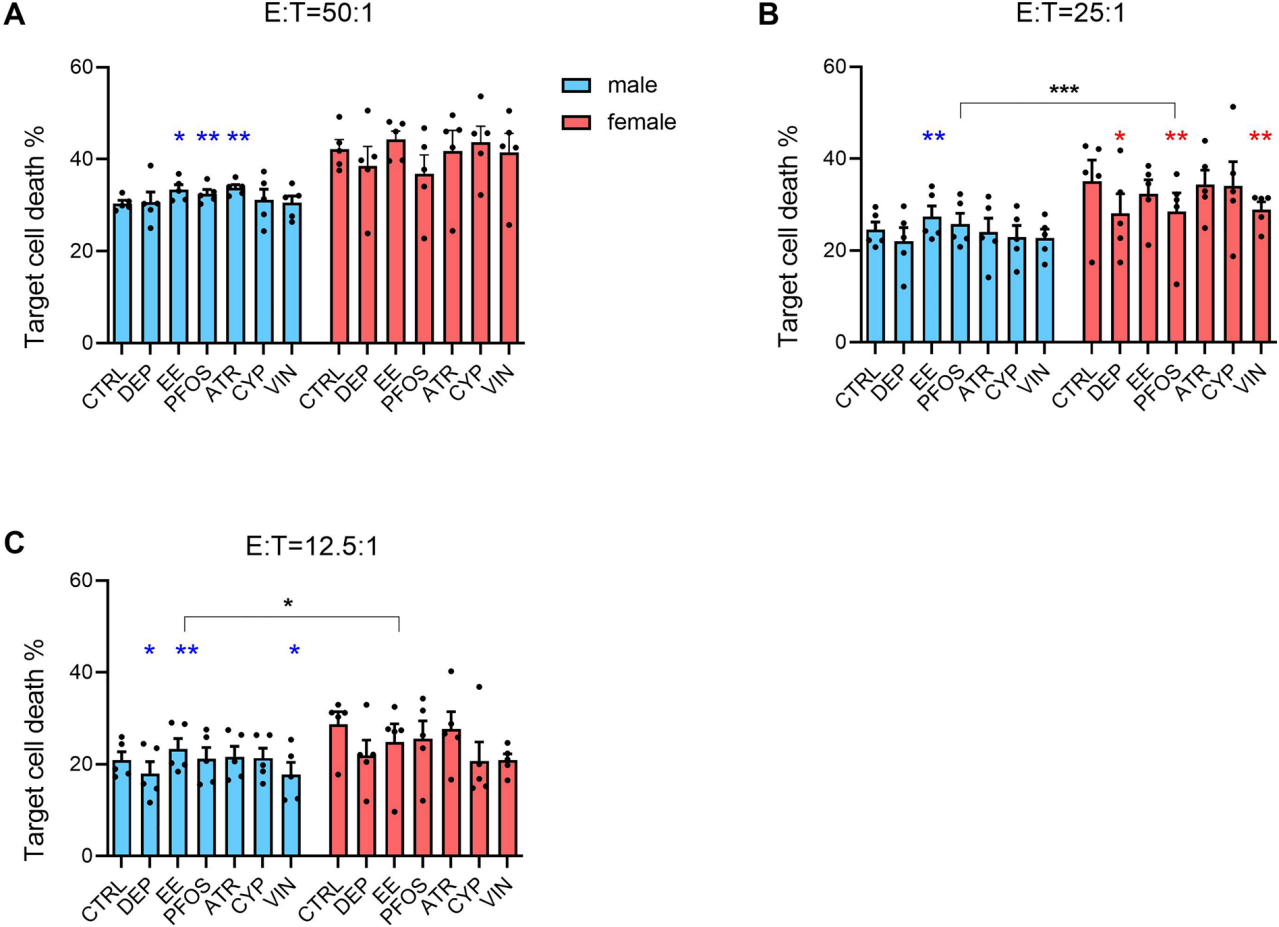
Effect of EDs on NK-cell activity. Male and female PBMC were exposed to the different EDs or DMSO (vehicle control) for 24 h and then co-cultured with K562 cells for 4 h at three different ratio between effector and target cells: 50:1 (**A**), 25:1 (**B**), and 12.5:1 (**C**). Target cell death was assessed by flow cytometry. Results are expressed as mean ± SEM of 5 male donors (light blue) and 5 female donors (pink) of % of dead cells (% of cells positive to PI staining within CFSE-positive cells). Each dot represents the value of the single donor. Statistical analysis was performed following paired *t* test, with *p ≤ 0.05, **p ≤ 0.01 *vs* CTRL (DMSO-treated PBMC co-cultured with K562 cells). Differences between males and females were assessed through unpaired *t* test, with *p ≤ 0.05, ***p ≤ 0.001 (color figure online)

NK cells are cytotoxic cells able to kill target cells, such as tumoral or virus-infected cells. PBMC, in which cells with NK activity are present, were treated with EDs for 24 h, and then co-cultured with CFSE-stained K562 cells (target cells) for 4 h (Fig. 3).

Different effector and target cell ratios were used: 50:1 (Fig. 3A), 25:1 (Fig. 3B), and 12.5:1 (Fig. 3C). Overall, only modest effects in NK-cell activity following EDs exposure were observed, most of them in males. The most effective compound was EE, which was able to increase male donors’ NK cells activity at all the tested effector:target ratios (Fig. 3A, 3B, C), with sex differences statistically significant at 12.5:1. Also PFOS exhibited sex differences in its action, specifically it was able to increase NK cells activity in males (Fig. 3A), and to decrease it in females (Fig. 3B). Minor effects were also observed in cells from healthy donors exposed to ATR, DEP, and VIN. DEP and VIN reduced NK-cell activity in both sexes, males at the lowest ratio and females at the middle one, respectively (Fig. 3B, C). Instead, ATR was able to slightly increase target cell death in male donors only at the highest ratio (Fig. 3A). With the exception of CYP, every ED was able to affect NK cells activity at least at one effector and target cell ratios, with sex differences observed for EE and PFOS.

To better express the lytic activity, integrating the three different ratios used, the results are also expressed as lytic unit 35 (LU_35_), meaning the ratio of effector cells necessary to kill the 35% of target cells. The LU_35_ are shown in Table 4. Both PFOS and ATR exposure in male donors resulted in a reduction in the lytic unit required to kill 35% of target cells, with respect to the control. Therefore, these EDs increased the lytic ability of male effector cells. Instead, in female donors, DEP and VIN induced an increase of LU_35_, reducing, therefore, the lytic ability of female effector cells. Regarding sex differences, it can be noted that DEP, EE, PFOS, and VIN show different trend based on donors’ sex, generally reducing LU_35_ in males and increasing them in females, meaning their ability to increase lytic activity in males, while reducing it in females.

**Table 4:** Lytic unit 35 (LU_35_) expressed as mean ± standard deviation

	Male donors	Female donors
CTRL	72.899 ± 12.661	25.408 ± 13.951
DEP	65.758 ± 17.242^#^	44.322 ± 25.416*^,#^
EE	56.495 ± 13.897^#^	29.888 ± 9.805^#^
PFOS	63.871 ± 13.576**^,#^	44.031 ± 28.870^#^
ATR	55.485 ± 8.834*	39.429 ± 36.992
CYP	94.498 ± 68.051	32.447 ± 19.383
VIN	71.162 ± 29.593^#^	44.245 ± 24.615*^,#^

The regression linear fit method was used to calculate LU_35_ for each condition. Statistical analysis was performed following paired *t* test, with

*p ≤ 0.05,

**p ≤ 0.01 *vs* CTRL, and differences between males and females were assessed through unpaired *t* test, with

^#^*p* ≤ 0.05

### ***Effects of EDs on CD4***^+^***and CD8***^+^***cells differentiation***

**Fig. 4:**
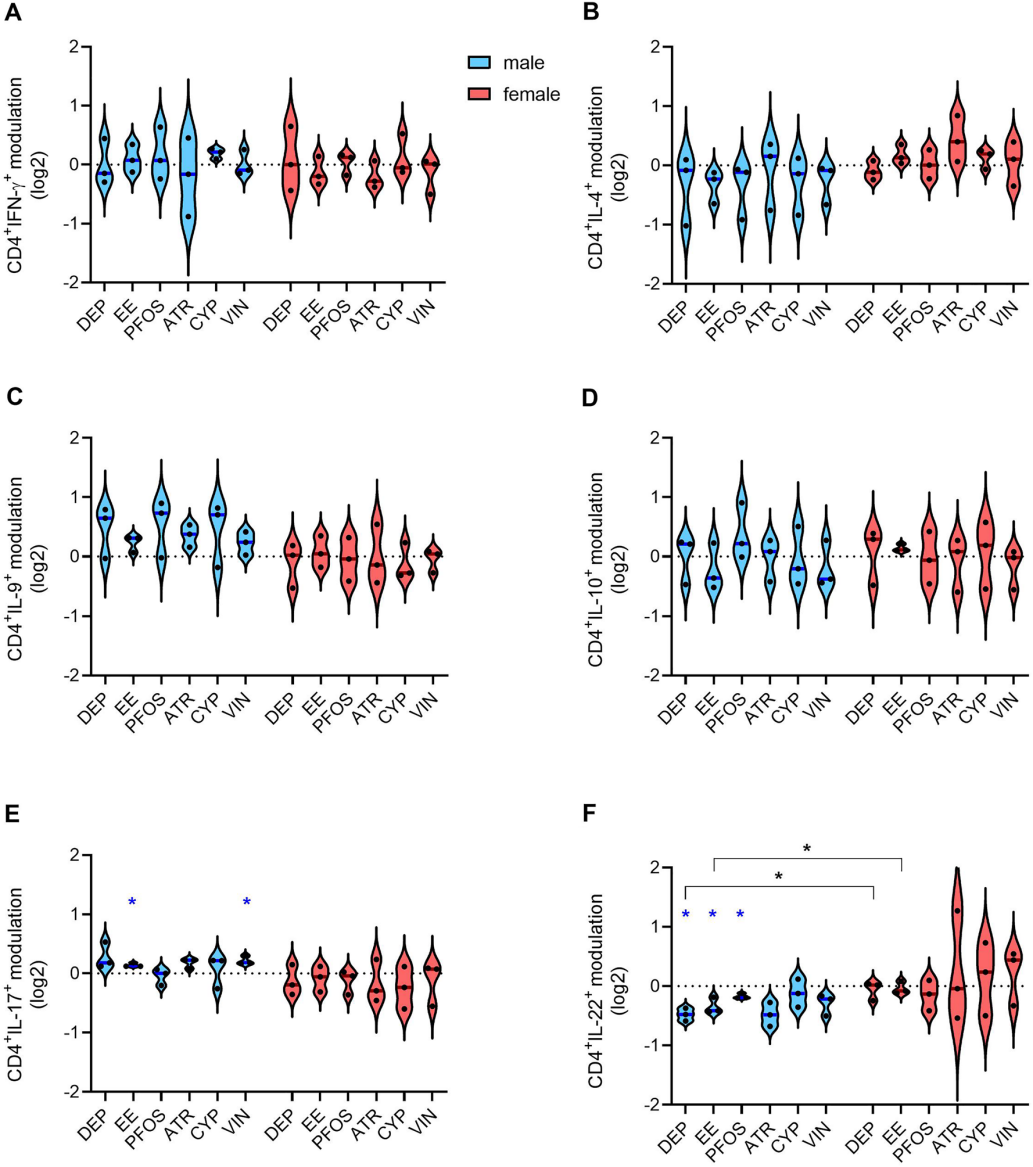
Modulation by EDs of cytokine-positive helper T cells. PBMC from males and females were exposed to the different EDs or DMSO (vehicle control) together with anti-CD3/anti-CD28-coated magnetic beads for 4 days. On gated CD4.^+^ lymphocytes, the cytokine-positive cells were identified by evaluating the expression of IFN-γ (**A**), IL-4 (**B**), IL-9 (**C**), IL-10 (**D**), IL-17 (**E**), and IL-22 (**F**) through flow cytometry. Results are shown as violin plots, with blue or red lines indicating the median fold modulation *vs* the respective DMSO-treated controls in 3 male (light blue) and 3 female (pink) donors, respectively. Each dot represents the cytokine modulation (log2 values) in a single donor. Statistical analysis was performed following unpaired t test with Welch’s correction, with *p ≤ 0.05 *vs* DMSO. Differences between males and females were assessed through unpaired *t* test (*p ≤ 0.05) (color figure online)

**Fig. 5:**
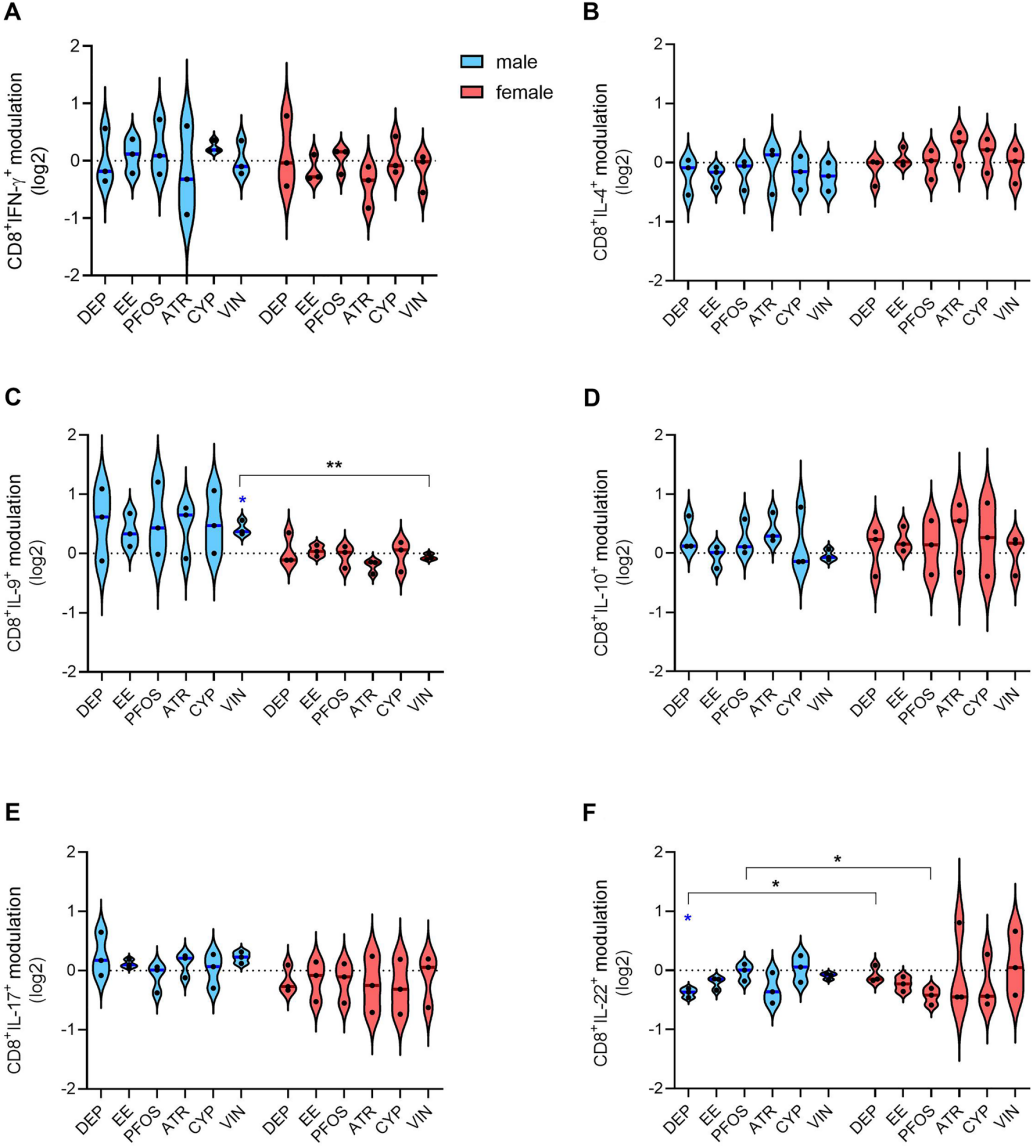
Modulation by EDs of cytokine-positive cytotoxic T cells. PBMC from males and females were exposed to the different EDs or DMSO (vehicle control) together with anti-CD3/anti-CD28-coated magnetic beads for 4 days. On gated CD8.^+^ lymphocytes, the cytokine-positive cells were identified by evaluating the expression of IFN-γ (**A**), IL-4 (**B**), IL-9 (**C**), IL-10 (**D**), IL-17 (**E**), and IL-22 (**F**) through flow cytometry. Results are shown as violin plots, with blue and red lines indicating the median fold modulation *vs* the respective DMSO-treated controls in 3 male (light blue) and 3 female (pink) donors, respectively. Each dot represents the cytokine modulation (log2 values) in a single donor. Statistical analysis was performed following unpaired *t* test with Welch’s correction, with *p ≤ 0.05 *vs* DMSO. Differences between males and females were assessed through unpaired *t* test (*p ≤ 0.05 and **p ≤ 0.01) (color figure online)

The main population of lymphocytes present in PBMCs are T lymphocytes. They can be divided into T helper (Th) cells and cytotoxic T cells, based on their expression of CD4 and CD8, respectively. Both populations can be further divided into subsets based on the expression of cytokines, transcription factors, and surface markers. In particular, within circulating T helper cells, Th1, Th2, Th9, Th17, Th22, and regulatory T (Treg) cells can be found. Following activation, the first 5 populations may be recognized by the expression of IFN-γ, IL-4, IL-9, IL-17, and IL-22, respectively. Whereas Treg can be recognized by the expression of CD25, GITR, FoxP3, and IL-10. However, conventional activated CD4^+^ cells are GITR^+^ and can express IL-10. The four-day activation with anti-CD3/CD28-coated magnetic beads induced activation, proliferation, and differentiation/polarization of CD4^+^ and CD8^+^ T cells present in PBMC and EDs exposure induced slight changes in CD4^+^ (Fig. 4) and CD8^+^ lymphocyte differentiation (Fig. 5).

Regarding CD4^+^ lymphocytes polarization, EE and VIN induced a slight increase in the percentage of cells CD4^+^IL-17^+^ in PBMC from male donors only (Fig. 4E). DEP, EE, and PFOS induced a decrease of the percentage of CD4^+^IL-22^+^ cells in PBMC from male donors, and a sex difference could be observed for DEP and EE (Fig. 4F). Instead, no significant modulation of the percentage of CD4^+^IFN-γ^+^, CD4^+^IL-4^+^, CD4^+^IL-9^+^, and CD4^+^IL-10^+^ cells has been observed (Fig. 4A, B, C, D).

While for T helper cells, the presence of different subpopulations is widely known and collectively accepted, for cytotoxic T cells only a few pieces of information are available, but they can be generally divided into Tc1, Tc2, Tc9, Tc17, and Tc22, similar to what occurs in T helper cells (Jiang et al. 2021; St Paul et al. 2021).

PBMC exposure to EDs induced some effects also regarding cytotoxic T-cell polarization. In particular, VIN increased the percentage of CD8^+^IL-9^+^ cells in PBMC from male donors only, with a statistical significance sex difference (Fig. 5C). Similarly to CD4^+^IL-22^+^ cells, DEP exposure resulted in a decreased percentage of CD8^+^IL-22^+^ cells in PBMC from male donors, with a sex difference, that could be observed also following PFOS exposure (Fig. 5F). Instead, no statistically significant effects were observed on CD8^+^IFN-γ^+^, CD8^+^IL-4^+^, CD8^+^IL-10^+^, and CD8^+^IL-17^+^ cells (Fig. 5A, B, D, E).

As previously mentioned, CD4^+^ Treg cells express FoxP3 and co-express CD25 and GITR and FoxP3 is a regulatory marker even in CD8^+^ cells. The role of CD25 and GITR in CD8^+^ cells is not well understood, but the expression of these molecules in cytotoxic T cells has been documented, and their involvement in immune regulation and immune tolerance promotion has been proposed (Ronchetti et al. 2012; Churlaud et al. 2015; Niederlova et al. 2021).

**Fig. 6:**
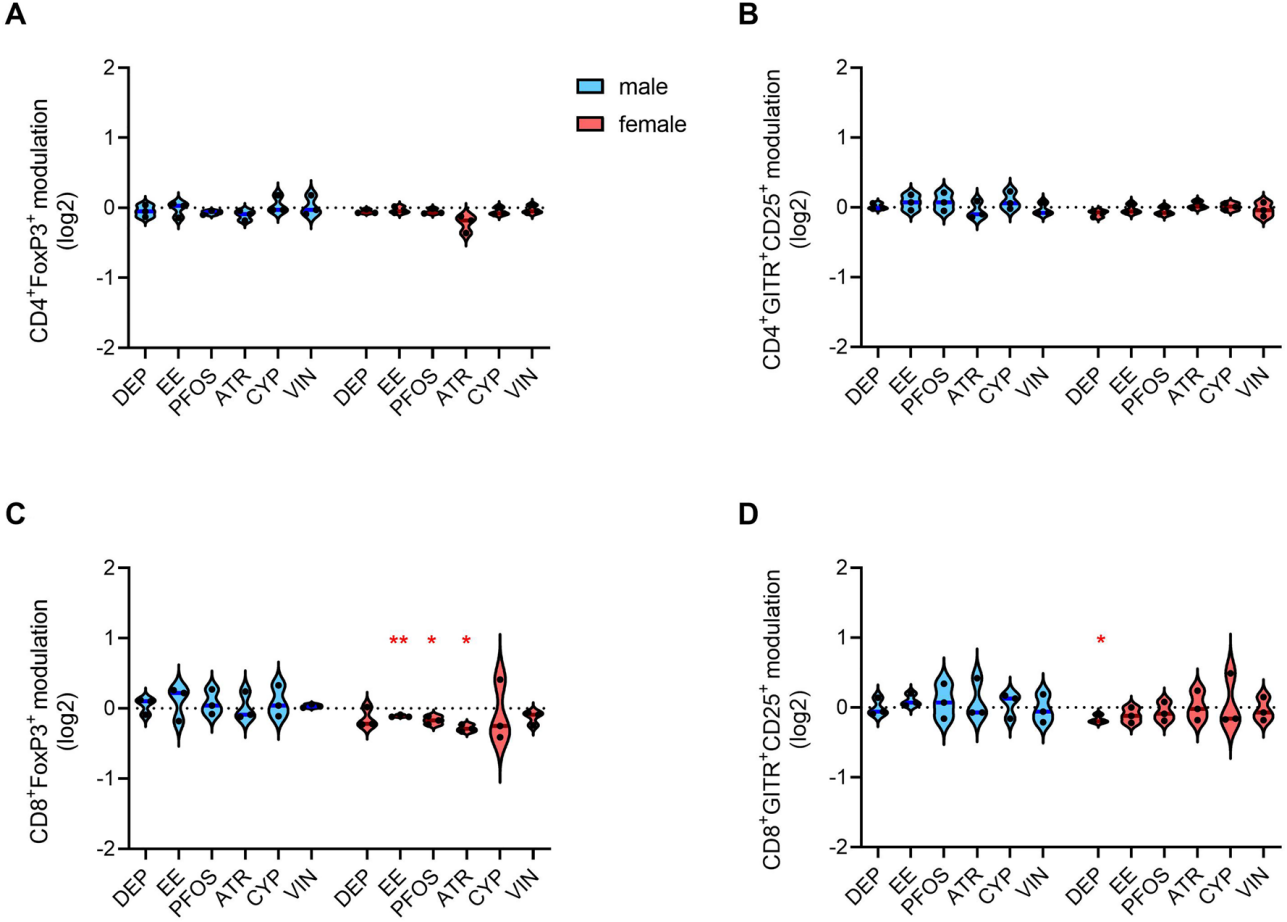
Modulation by EDs of cells expressing Treg-related markers. PBMC from males and females were exposed to the different EDs or DMSO (vehicle control) together with anti-CD3/anti-CD28-coated magnetic beads for 4 days. On gated CD4^+^ (**A**, **B**) and CD8^+^ (**C**, **D**) lymphocytes, FoxP3-positive (**A**, **C**) and GITR-CD25 double-positive (**B**, **D**) cells were identified. Results are shown as violin plots, with blue and red lines indicating the median fold modulation *vs* the respective DMSO-treated controls in 3 male (light blue) and 3 female donors (pink), respectively. Each dot represents the marker modulation (log2 values) in a single donor. Statistical analysis was performed following unpaired *t* test with Welch’s correction, with *p ≤ 0.05 and **p ≤ 0.01 *vs* DMSO (color figure online)

Regarding CD4^+^ Treg cells, no effects of the selected EDs have been observed (Fig. 6A, B). Instead, EE, PFOS, and ATR induced a modest decrease of CD8^+^FoxP3^+^ cells in female donors (Fig. 6C), and DEP induced a slight decrease of CD8^+^GITR^+^CD25^+^ cells in female donors (Fig. 6D). Therefore, a modest decrease of CD8^+^ Treg in female donors due to DEP, EE, PFOS, and ATR could be observed.

### ***Sex differences in EDs’ modulations of CD4***^+^***subpopulations***

Using the canonical elaboration of flow cytometric, data suggested that EDs had few effects on T-cell differentiation/polarization. Since it seemed unlikely, a more in-depth analysis was conducted using the t-SNE algorithm that groups cells in subsets, based on the different expression levels of the stained markers. To do this, we divided the EDs into two groups, highly contaminating EDs (DEP, EE, and PFOS) and pesticides (ATR, CYP, and VIN).

**Fig. 7:**
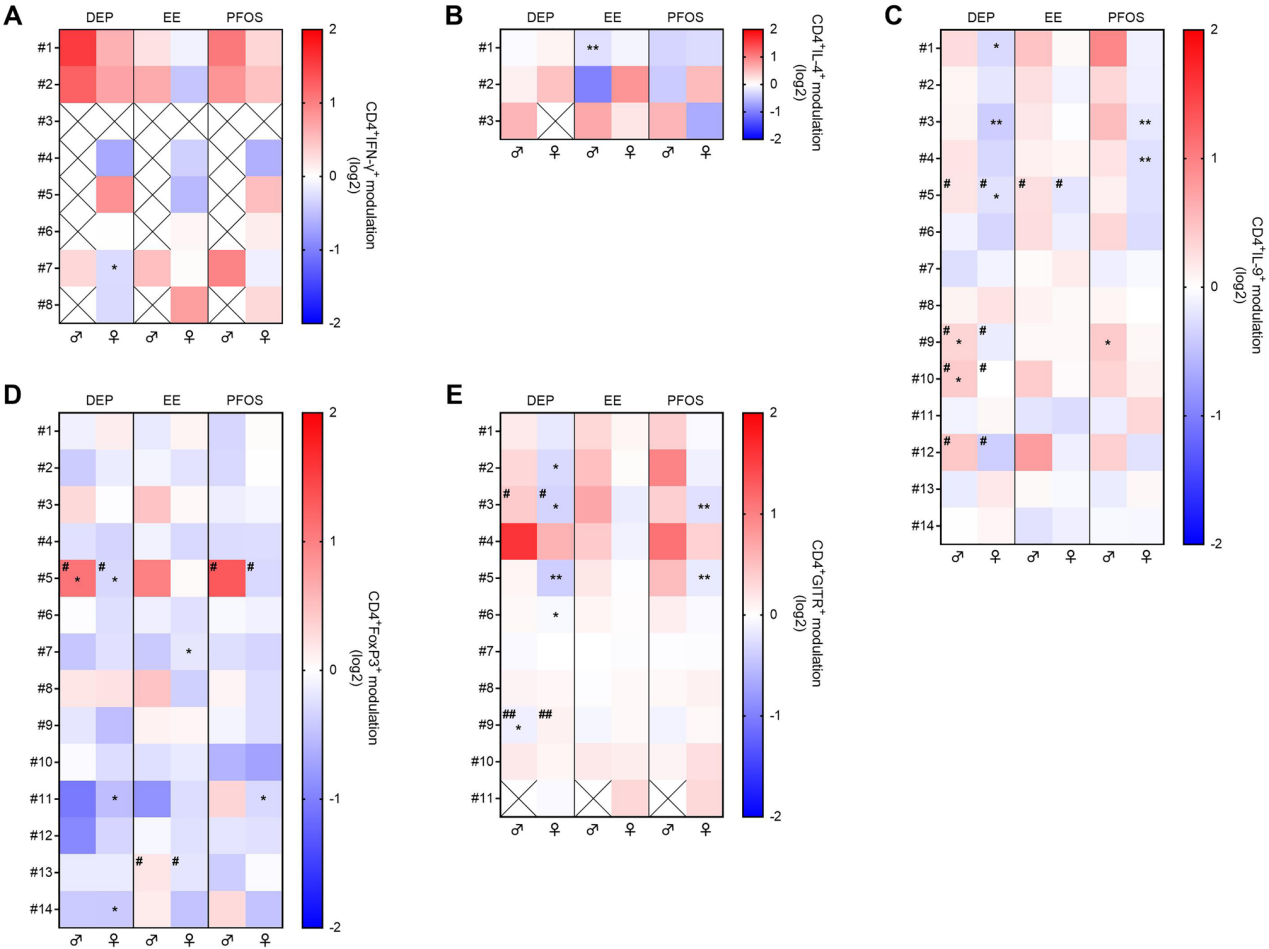
Modulation by DEP, EE, and PFOS of CD4^+^ cell subsets, evaluated through t-SNE analysis. Male (♂) and female (♀) PBMC were exposed to the EDs or DMSO (vehicle control) together with anti-CD3/anti-CD28-coated magnetic beads for 4 days. After gating CD4.^+^ cells, subsets were identified based on staining with eleven antibodies as reported in the Material and Methods section. Cell subsets expressing high levels of IFN-γ (**A**), IL-4 (**B**), IL-9 (**C**), FoxP3 (**D**), and GITR (**E**) are numbered. The cell percentage of each treated donor was divided by the cell percentage of the same DMSO-treated donor (0, white) and expressed as log2 (modulation ratio). The mean modulation ratio of male and female donors is reported in the double gradient heatmap (red, increase; blue, decrease). White squares with the cross indicate that the subset of cells is absent in at least one DMSO-treated donor so the modulation ratio cannot be evaluated. Statistical analysis was performed following unpaired *t* test with Welch’s correction, with *p ≤ 0.05 and **p ≤ 0.01 *vs* DMSO-treated PBMCs. Differences between males and females were assessed through unpaired *t* test (#*p* ≤ 0.05 and ##*p* ≤ 0.01) (color figure online)

By considering data from the 6 donors following DEP, EE, PFOS exposure, the t-SNE analysis found 50 relevant subsets expressing high levels of one marker in CD4^+^ cells: 8 IFN-γ^+^ subsets, 3 IL-4^+^ subsets, 14 IL-9^+^ subsets, 14 FoxP3^+^ subsets, and 11 GITR^+^ subsets. Some of these subsets express more than one marker, as can be observed in Supplementary Fig. 6. The mean percentage of cells in each subset after treatment is divided by that in the control solvent for males and females and the values are reported in Fig. 7. The mean cell percentage of several subsets was modulated. In males, the percentage of cells present in subset #1 of IL-4^+^ cells was decreased following EE exposure. Whereas, DEP exposure in general increased the percentage of IL-9^+^ cells (mainly #9 and 10) and of FoxP3^+^ cells (#5), while decreased subset #9 of GITR^+^ cells. Also PFOS increased subset #9 of IL-9^+^ cells. In females, instead, more modulations were observed. In detail, EE reduced the percentage of cells present in subset #7 of FoxP3^+^ cells. DEP exposure reduced the percentage of cells present in subset #7 of IFN-γ^+^ cells, reduced the percentage of IL-9^+^ cells (#1, 3, and 5), of FoxP3^+^ cells (#5, 11, and 14), and of GITR^+^ cells (#2, 3, 5, and 6). PFOS also reduced the percentage of IL-9^+^ cells (#3 and 4), #11 of FoxP3^+^ cells, and #3 and 5 of GITR^+^ cells. Even more interestingly, neither DEP, nor PFOS, nor EE had the same effects in females and males in each subset. In particular, looking at the subsets of cells positive to IL-9 and GITR (Fig. 7C and E) a general increase of the subsets in males and a decrease in females can be observed, and significant differences in the modulation in males *vs* females were observed. Subsets #5, 9, 10, and 12 of IL-9^+^ cells were differently modulated by DEP based on the sex, and subset #5 also by EE exposure (Fig. 7C). Also, several subsets of FoxP3^+^ cells were altered differently based on the sex; #5 by DEP and PFOS exposure, and #13 by EE (Fig. 7D). Regarding GITR^+^ cells, subsets #3 and 9 were differently modulated based on the sex (Fig. 7E). Overall, a clear sex bias in the effects induced by DEP, PFOS and EE exposure is observed. For those populations statistically significantly altered by EDs exposure, the log2 value of the modulation, together with the abundance of the population within CD4^+^ cells is reported in Supplementary Table 1.

**Fig. 8:**
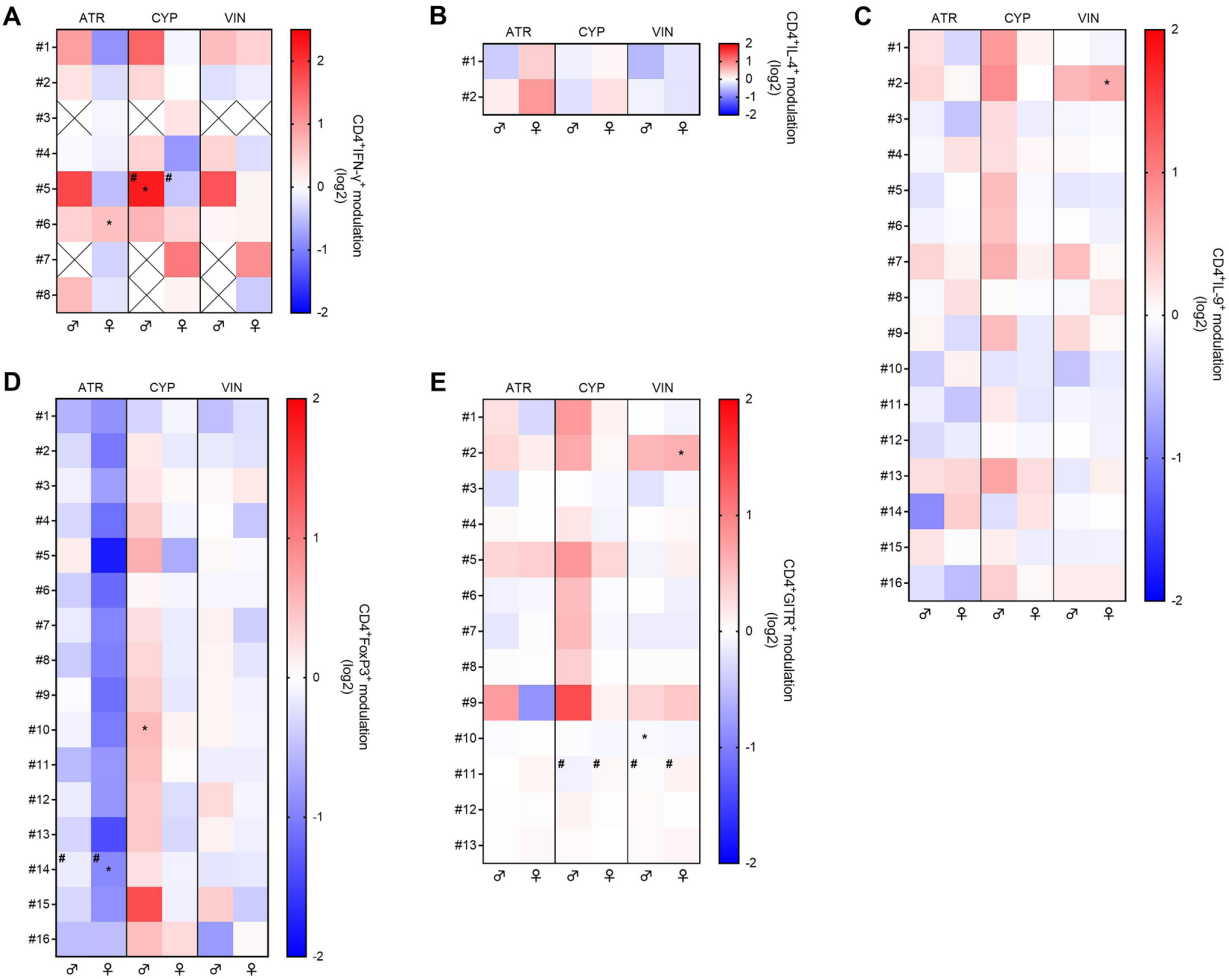
Modulation by ATR, CYP, and VIN of CD4^+^ cell subsets, evaluated through t-SNE analysis. Male (♂) and female (♀) PBMC were exposed to the EDs or DMSO (vehicle control) together with anti-CD3/anti-CD28-coated magnetic beads for 4 days. After gating CD4.^+^ cells, subsets were identified based on staining with eleven antibodies as reported in the Material and Methods section. Cell subsets expressing high levels of IFN-γ (**A**), IL-4 (**B**), IL-9 (**C**), FoxP3 (**D**), and GITR (**E**) are numbered. The cell percentage of each treated donor was divided by the cell percentage of the same DMSO-treated donor (0, white) and expressed as log2 (modulation ratio). The mean modulation ratio of male and female donors is reported in the double gradient heatmap (red, increase; blue, decrease). White squares with the cross indicate that the subset of cells is absent in at least one DMSO-treated donor so the modulation ratio cannot be evaluated. Statistical analysis was performed following unpaired *t* test with Welch’s correction, with *p ≤ 0.05 *vs* DMSO-treated PBMCs. Differences between males and females were assessed through unpaired *t* test (#*p* ≤ 0.05) (color figure online)

Regarding the analysis including solvent control and treatment with ATR, CYP, and VIN in CD4^+^ cells, the t-SNE analysis revealed 55 subsets expressing high levels of one marker: 8 IFN-γ^+^ subsets, 2 IL-4^+^ subsets, 16 IL-9^+^ subsets, 16 FoxP3^+^ subsets, and 13 GITR^+^ subsets were retrieved. Some of these subsets express more than one marker, as can be observed in Supplementary Fig. 7. The mean percentage of cells in each subset after treatment is divided by that in the control for males and females and the values are reported in Fig. 8. The mean cell percentage of some subsets was modulated in males or females. Also, the effects of ATR, CYP, and VIN showed sex-bias effects but the tendency was less evident. In males, the percentage of IFN-γ^+^ cells (#5) was increased following CYP exposure and also subset #10 of FoxP3^+^ cells. VIN exposure to male donors induced a slight increase of subset #10 of GITR^+^ cells. In females, ATR was able to increase the percentage of cells positive to IFN-γ present in subset #6, and to decrease the percentage of cells present in subset #14 of FoxP3^+^ cells. VIN exposure to female donors, instead, statistically significantly increased the percentage of IL-9^+^ cells (#2) and of GITR^+^ cells (#2). Differently, from the three highly persistent EDs, the three pesticides here analyzed do not show a strong difference between male and female donors. The only statistical differences were observed on subset #5 of IFN-γ^+^ cells following CYP exposure, which increased the percentage of cells in males and decreased it in females (Fig. 8A), and on subset #14 of FoxP3^+^ cells, where ATR decreased the percentage of cells in both sexes but with a greater effect on females (Fig. 8D). Similarly to the other EDs, the log2 values of the modulation relative to the populations statistically significantly altered by ATR, CYP, and VIN exposure are reported in Supplementary Table 2.

**Fig. 9:**
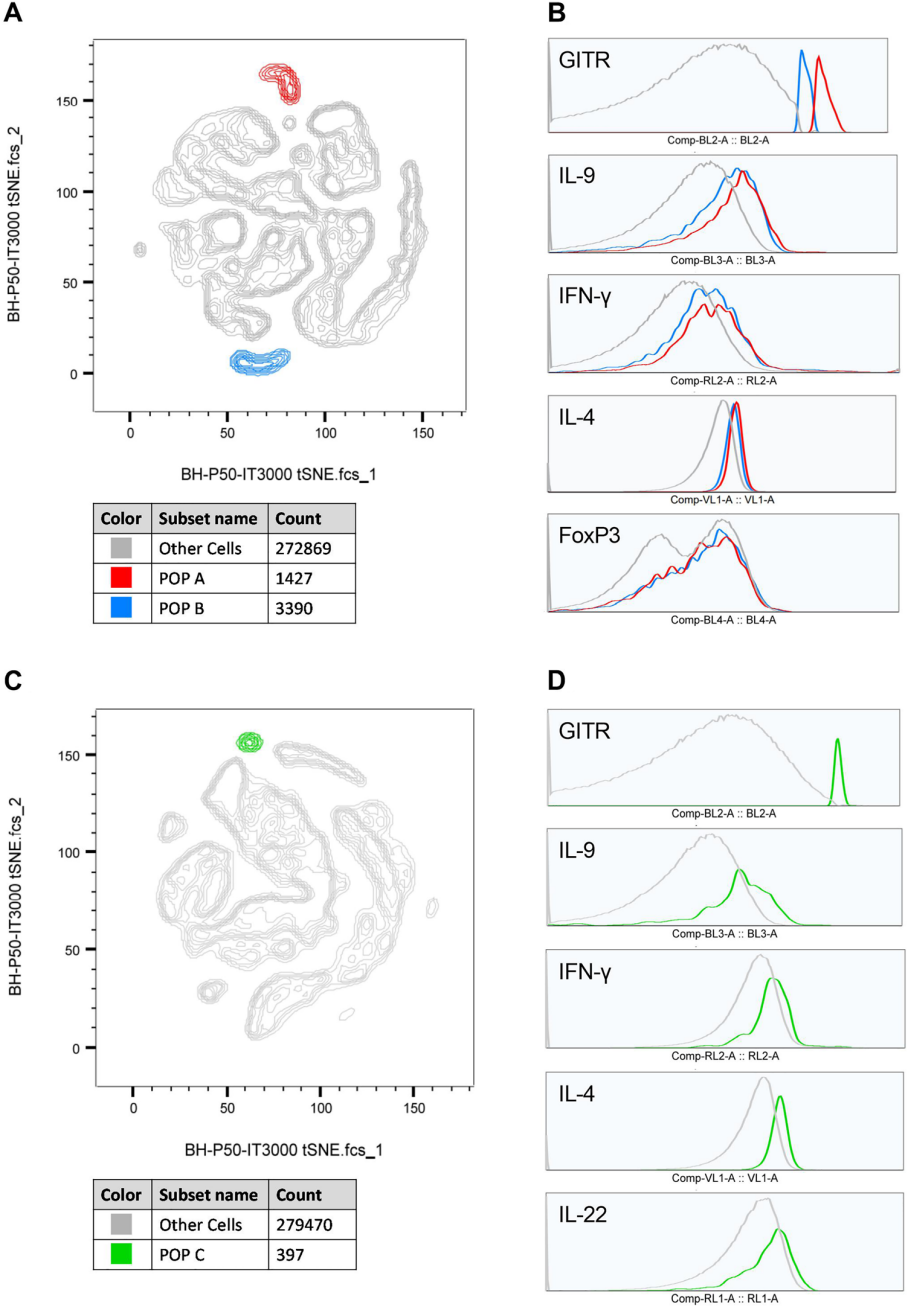
2D t-SNE maps representing the most interesting subsets of population modulated by the selected EDs, which share multiple markers (**A**, **C**). Population A and B are referred to the t-SNE plot of DEP, EE, and PFOS (**A**), whereas Population C is referred to the t-SNE plot of ATR, CYP, and VIN (**C**). Population A (POP A—red) is represented by cells present in the subsets #7 of IFN-γ, #1 of IL-9, #5 of FoxP3, and #2 of GITR (Supplementary Fig. 6) and they are reduced in a statically significant way by DEP exposure in female donors. Population B (POP B – blue) is represented by cells present in the subsets #8 of IFN-γ, #3 of IL-9, #11 of FoxP3, and #5 of GITR (Supplementary Fig. 6) and they are reduced in a statically significant way by both DEP and PFOS exposure in female donors. Population C is represented by cells contained in the subsets #2 of IL-9 and #2 of GITR (Supplementary Fig. 7) and they are increased in a statically significant way by VIN exposure in female donors. Note that it was not possible to find POP C also in the other markers because these populations were not present in all the conditions of exposure and not in all donors. The phenotypes of POP A, B, and C are reported (B, D), where the gray line represents the phenotype of the other cells. The other phenotypes (all 11 markers) are shown in Supplementary Fig. 8 (color figure online)

By overlapping the different 2D t-SNE projection maps obtained from the exposure to DEP, EE, and PFOS we realized that the cells in subset #7 of IFN-γ are the same as subset #1 of IL-9, #5 of FoxP3, and #2 of GITR (Fig. 7 and Supplementary Fig. 6) and called the subset POP A (Fig. 9A). Moreover, the cells in the subset #8 of IFN-γ are present also in subset #3 of IL-9, #11 of FoxP3, and #5 of GITR (Fig. 7 and Supplementary Fig. 6) and called the subset POP B (Fig. 9A). POP A and B are significantly decreased by DEP exposure in female donors only. POP B is significantly decreased also by PFOS exposure in female donors only. POP A and B are both CD4^+^FoxP3^+^GITR^+^IFN-γ^+^IL-4^+^IL-9^+^ with POP A expressing more GITR than POP B (Fig. 9B, Suppl Fig. 8A). The expression of GITR together with the cytokines may indicate that the cells of the subset belong to activated conventional CD4^+^ T cells. However, the expression of FoxP3 may indicate that the cells of the subset act as regulatory T cells.

Lastly, analyzing the 2D t-SNE maps obtained from ATR, CYP, and DEP exposure, we discovered the subset POP C (Fig. 9C) which cells are present in subset #2 of IL-9 and #2 of GITR (Fig. 8 and Supplementary Fig. 7). POP C is significantly increased by VIN exposure in both sexes, but the increase is significant only in woman donors. The phenotype of this population is CD4^+^IL-22^+^IL-4^+^IFN-γ^+^IL-9^+^GITR^+^, with a very high expression of GITR compared to the not affected population (Fig. 9D, Suppl Fig. 8B). Therefore, the main cytokines characterizing this population, which is increased by VIN exposure, mainly in women, are IFN-γ, IL-4, IL-9. The high level of expression of GITR may indicate that the cells of the subset belong to activated conventional CD4^+^ T cells. The complete phenotypes can be retrieved in Supplementary Fig. 8.

## Discussion

In the last decades, an increase in several diseases, namely tumors, obesity, psychiatric disorders, and autoimmunity, has been observed, above all in developed countries. Within the main causes, environmental factors, including EDs, are considered the main responsible (Manley et al. 2018). Exposure to EDs has been related to diseases that involve, directly or indirectly, the immune system, such as inflammatory disorders, allergy, asthma, autoimmunity, obesity, type 2 diabetes, and cancer (Schug et al. 2011; Teitelbaum et al. 2012; Bekö et al. 2013; Bertelsen et al. 2013; Trasande et al. 2013; Buser et al. 2014; Schooling and Zhao 2015; Benvenga et al. 2020; Predieri et al. 2020; Segovia-Mendoza et al. 2020; Schjenken et al. 2021). The majority of these disorders are characterized by a sex dimorphism, meaning a different prevalence or susceptibility, different onset, progression, severity, survival, or response to therapy of the two sexes (Ortona et al. 2016; Selmi and Gershwin 2019; Di Florio et al. 2020; Klein and Morgan 2020; Massey et al. 2021). For example, autoimmune disorders usually affect more women than men (Quintero et al. 2012; Angum et al. 2020). Steroid hormones, such as sex hormones and corticosteroids, are known to interact with the immune system, leading to a sexual dimorphism that involves the endocrine, nervous, and immune systems (Gaillard and Spinedi 1998; Bhatia et al. 2014; Taneja 2018). Regarding the immune system, women’s immune system is considered more reactive, and therefore less susceptible to infections but more prone to develop several immune disorders, like autoimmunity or exaggerated immune responses (Butterworth et al. 1967; Mangalam et al. 2013; Ngo et al. 2014). The interaction between steroid hormones and environmental factors can provoke different immune responses based on gender (Sugiyama et al. 2010; Ghosh and Klein 2017). When considering the entire organisms, also the impact of microbiota must be mentioned due to the strict interconnection with the endocrine system (Mayer et al. 2015; Park and Choi 2017; Qi et al. 2021). Therefore, due to the wide variety of factors that lead to a sex dimorphism in health and diseases, it is important to study the adverse effects of substances in both sexes, above all in the case of EDs.

In our study, we demonstrated that the selected EDs exerted adverse immune effects in vitro in both male and female PBMCs. Indeed, modulation of RACK1 was induced by all the tested EDs at least in one sex at the tested concentrations. Some of them also resulted in the modulation of the pro-inflammatory response, more specifically DEP, PFOS, ATR, and CYP induced a reduction of the pro-inflammatory response, which reflects the decreased RACK1 expression. These results confirm previous evidence of the modulation of immune parameters by EDs on cell lines (Maddalon et al. 2022; Masi et al. 2022). Regarding sex dimorphism, female donors seem to be more susceptible to DEP reduction of pro-inflammatory cytokines. Also EE induced different effects on RACK1 expression based on the sex, similar to what is observed on the impact on NK cells’ lytic activity. On these cells also PFOS show a different effect, resulting in an increased lytic ability in males and a decreased one in females. Previous evidence showed the ability of PFOS to reduce NK activity in mice offspring following gestational exposure (Keil et al. 2008). All the other tested chemicals, with the exception of CYP, resulted in an impairment of NK activity, indicating their ability to modulate the immune system in vitro. NK activity decrease by VIN confirms what is already present in the literature (White et al. 2004). Regarding T-cell differentiation, EE and VIN exposure resulted in a slightly increased percentage of IL-17-producing cells (likely Th17), while DEP, EE, and PFOS exposure decreased the percentage of IL-22-producing cells (likely Th22), with DEP and EE evidencing a higher activity in males *vs* females. Similarly, also Tc22 percentage resulted decreased in males upon DEP exposure, highlighting also in this case a higher men susceptibility. Finally, VIN increased Tc9 in male donors, differently from what was observed in women’s cells. Regarding the effects observed in women, the percentage of CD8^+^FoxP3^+^ cells resulted to be reduced upon EE and mainly PFOS and ATR exposure, and DEP slightly reduced CD8^+^GITR^+^CD25^+^ cells.

The deeper analysis of T helper cells subpopulations conducted by t-SNE evidenced other immunomodulation by EDs, with sex bias in several results, above all in the general modulation of Th1, Th2, Th9, and GITR^+^ cells, mainly upon DEP and PFOS exposure. DEP’s ability to reduce IL-4 and IFN-γ production was already demonstrated in vitro but without considering the possible sex bias (Hansen et al. 2015). Also for PFOS, there are evidence of its ability to perturb the balance between Th1 and Th2 in mice (Dong et al. 2011; Zheng et al. 2011; Yang et al. 2021).

We observed the most relevant sex bias in DEP-treated samples with seven subpopulations demonstrating a significant sex bias and other subpopulation showing different DEP-dependent modulation in females and males, thought non-significant. In particular, DEP determined decrease of 11 subpopulations in females and increase of 3 subpopulations in males. The relevant DEP-dependent decrease of POP A and B is of particular interest (Fig. 7) because these subpopulations represent about 0.5% and 1.2% of CD4^+^ T cells (Fig. 9, panel A), respectively. They express FoxP3 at high levels, but, in our experimental setting, about half of CD4^+^ cells express FoxP3 (Fig. 9, panel B), suggesting that here, as in other experimental models, FoxP3 is expressed more by activated T cells than regulatory T cells (Wang et al. 2007). POP A and B are also characterized by high expression of GITR. GITR (TNFRSF18), originally described as induced by glucocorticoid in a T-cell line, is mainly expressed in those active lymphocytes involved in immune tolerance and also in conventional T lymphocytes following activation (Placke et al. 2010; Ronchetti et al. 2015; Nocentini et al. 2017; Riccardi et al. 2018). Moreover, POP A and B express quite high levels of IFN-γ, IL-4 and IL-9, suggesting that they represent activated conventional T cells with a peculiar phenotype. Thus, the DEP-dependent decrease of POP A and B in females (Fig. 7) would suggest an immunosuppressive effects of DEP in females in agreement with the DEP-dependent downmodulation of RACK1, CD86, CD54, IL-8 and TNF-α expression (Figs. 1 and 2) and the decrease of NK-cell activity.

Interestingly, t-SNE analysis suggests that the activity of DEP and the other EDs is specific, having modulatory effects in some subpopulations of lymphocytes, whose functional meaning need to be investigated by dose–response in vitro and in vivo studies. For example, the PFOS-dependent decrease of POP B in males is somehow counteracted by the PFOS-dependent increase of POP A, suggesting a specific fine tuning of EDs on immune system of males and females. POP C (Fig. 9, panels C–D), a subpopulation very similar to POP A and B, is another example of fine tuning. In fact, it is significantly increased by VIN in females but not in males (Fig. 8). Finally, the relevant decrease of FoxP3^+^ cells following ATR treatment is relevant in almost all subpopulations in females (significant in #14) and irrelevant or absent in males (Fig. 8, panel D). Interestingly, we previously demonstrated the ability of DEP and PFOS to interact with the glucocorticoid receptor as agonists (Masi et al. 2022). Regarding VIN, its M2 metabolite (3′,5′-dichloro-2-hydroxy-2-methylbut-3-enanilide) showed a weak antagonism toward the glucocorticoid receptor (Molina-Molina et al. 2006). Therefore, there could be a possible explanation of the relationship between DEP, PFOS, and VIN with GITR expression.

POP A, B, and C are characterized also by high expression of IFN-γ, IL-4, and IL-9. A population of T helper cells co-expressing IL-9 and IL-4 was already reported in the literature, indicating that this population can activate eosinophils in colitis and is involved in the effector function of T helper cells in this disease (Moshkovits et al. 2017; Stanko et al. 2018). IFN-γ^+^IL-4^+^ double-positive cells have been also described (Krawczyk et al. 2007). Furthermore, IL-9 can be also produced by Treg (Lu et al. 2006). Although Th1, Th2, and Th9 exhibit different T-cell phenotypes, some gene clusters are similarly regulated (Xue et al. 2019). More in-depth investigation to discover the involvement of these cell subsets in EDs immunomodulatory effects must be performed.

The similar action between DEP and PFOS could be also explained by their common involvement in the estrogenic pathway (Du et al. 2013; Fiocchetti et al. 2021), and this could also represent a possible explanation for the sex bias. They both reduced RACK1 expression in both sexes, with a parallelism with immunological implications (reduced pro-inflammatory markers). They also reduced the percentage of CD4^+^IL-22^+^ cells and modulated CD4^+^IL-9^+^, CD4^+^FoxP3^+^, and CD4^+^GITR^+^ similarly.

Glucocorticoids are considered immunosuppressors, since they inhibit several immune cell activities (Van Laethem et al. 2001; Strehl et al. 2019). For example, they are able to suppress T-cell activation and NK-cell activity (Muscari et al. 2022), which is in line with the effects observed with PFOS and DEP exposure, both substances able to activate glucocorticoid receptor. Regarding T helper cells differentiation, glucocorticoids are able to suppress T helper cells, together with their effector functions (Liberman et al. 2018; Strehl et al. 2019; Taves and Ashwell 2021). In general, all CD4^+^ T cells are sensitive to glucocorticoid-induced inhibition, like Th1, Th2, Th9, and Th22 (Arya et al. 1984; Wu et al. 1991; Holz et al. 2005; Cao et al. 2012), with the only exception of Th17 and Treg (Banuelos and Lu 2016; Cari et al. 2019). Also in this case, this is in line with the reduction of POP A and B exerted by DEP and PFOS in female donors, populations characterized by highly expression of cytokines representative of Th1, Th2, and Th9.

Due to the wide importance of the immune system and sex dimorphism in diseases, it is important to study the effect of EDs on the immune system, focusing on the possible sex difference. Our study provides an overview of the effects of EDs on the immune system, mainly focusing on PBMCs and lymphocytes. To confirm these data, more subjects should be tested, but since we already observed effects, it is presumable to find even more with more samples. We have also tested only one concentration of each ED. This allowed us to test and compare more substances at concentrations relevant to human exposure, but a wider range of concentrations should also be tested for a broader view. Therefore, with our in vitro study testing the effects of 6 EDs on primary immune cells of both sexes, we can affirm the ability of EDs to modulate the immune system, both innate and adaptive response. In detail, they modulated pro-inflammatory activity, natural killer lytic ability, and lymphocyte activation and differentiation with different effects. In particular, DEP and PFOS appeared to be the two high concern EDs, within the ones selected in this study. Therefore, more studies focusing on them should be performed.

### Supplementary Information

Below is the link to the electronic supplementary material.

Supplementary file1 (DOCX 2682 KB)

## Data Availability

All the data that support the findings of this study are available from the corresponding author upon reasonable request.
