# Supplementary material for: Impact of endocrine disruptors on peripheral blood mononuclear cells in vitro: role of gender
Source: Arch Toxicol. 2023 Sep 7;97(12):3129–50. doi: 10.1007/s00204-023-03592-3 (PMC10567873; doi:10.1007/s00204-023-03592-3)
Supplement: Supplementary file 1 — Supplementary file1 (DOCX 2682 KB) [file 204_2023_3592_MOESM1_ESM.docx]

# *Supplementary Material*

**Supplementary Figure 1.**

**
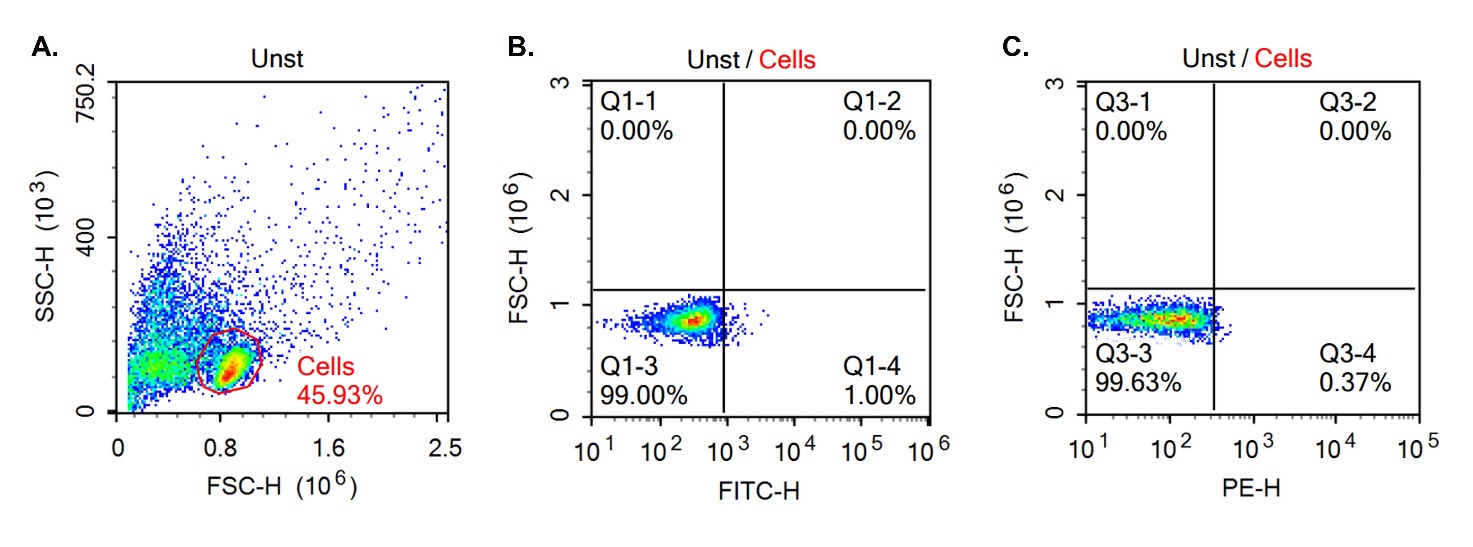
**

**Supplementary Figure 1 – Gating strategy for peripheral blood mononuclear cells for flow cytometry.** Hierarchical gating to identify cells positive to CD86 (FITC) and CD54 (PE). a) Representative dot plot diagram of forward-scatter (FSC) and side-scatter (SSC) light. Cells gate (red gate) was created to highlight the main lymphocyte population. b) Representative dot plot diagram of CD86^+^ cells (Q1-4) inside Cells gate (red gate). c) Representative dot plot diagram of CD54^+^ cells (Q3-4) inside Cells gate (red gate). Plots are for illustration purposes only.

**Supplementary Figure 2.**

**
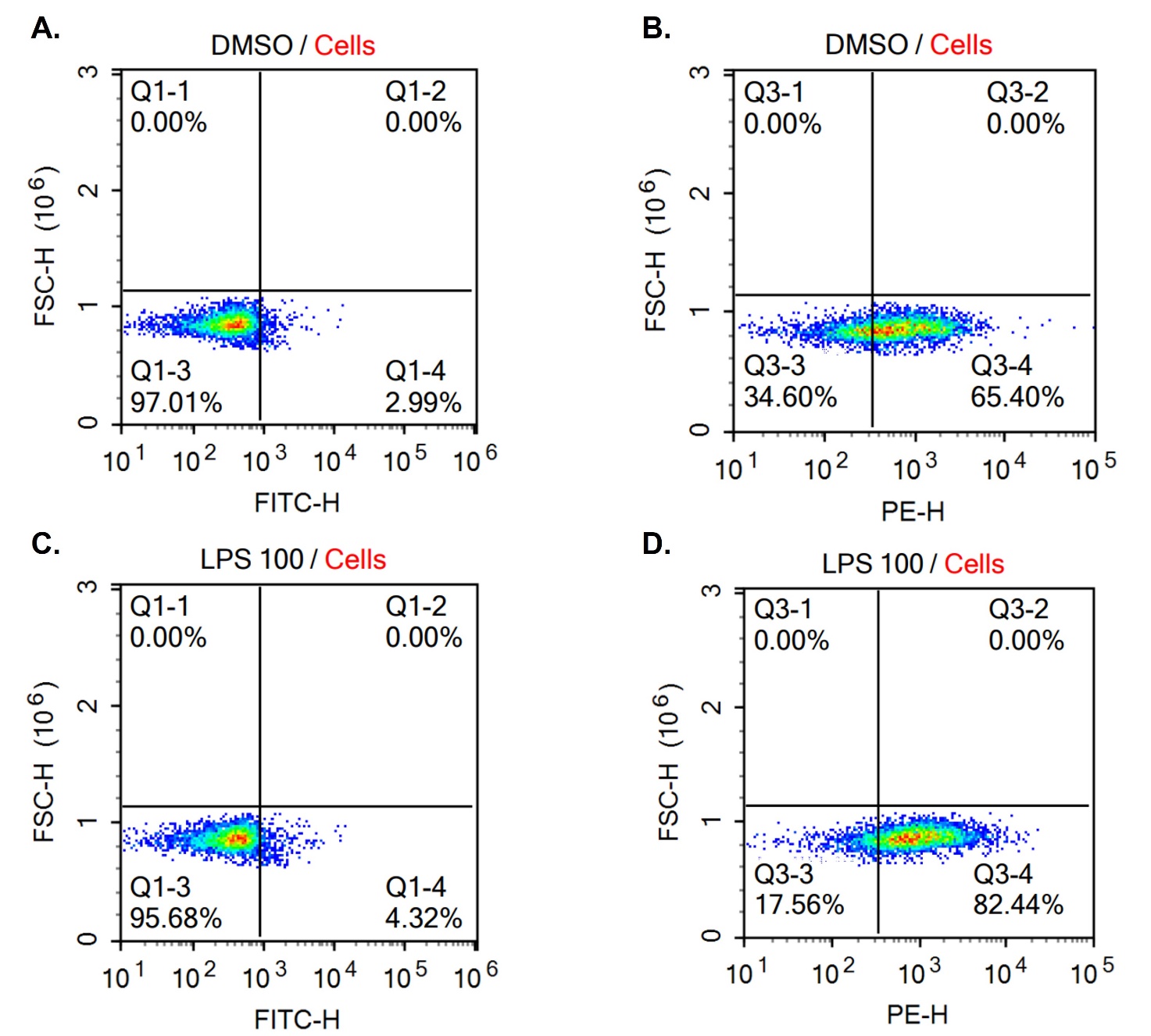
**

**Supplementary Figure 2 – Representative dot plots of DMSO- and LPS 100 ng/ml-treated peripheral blood mononuclear cells.** a) Representative dot plot diagram of CD86^+^ DMSO-treated cells (Q1-4). b) Representative dot plot diagram of CD54^+^ DMSO-treated cells (Q3-4). Plots are for illustration purposes only. c) Representative dot plot diagram of CD86^+^ LPS-treated cells (Q1-4). d) Representative dot plot diagram of CD54^+^ LPS-treated cells (Q3-4). Plots are for illustration purposes only.

**Supplementary Figure 3.**

**
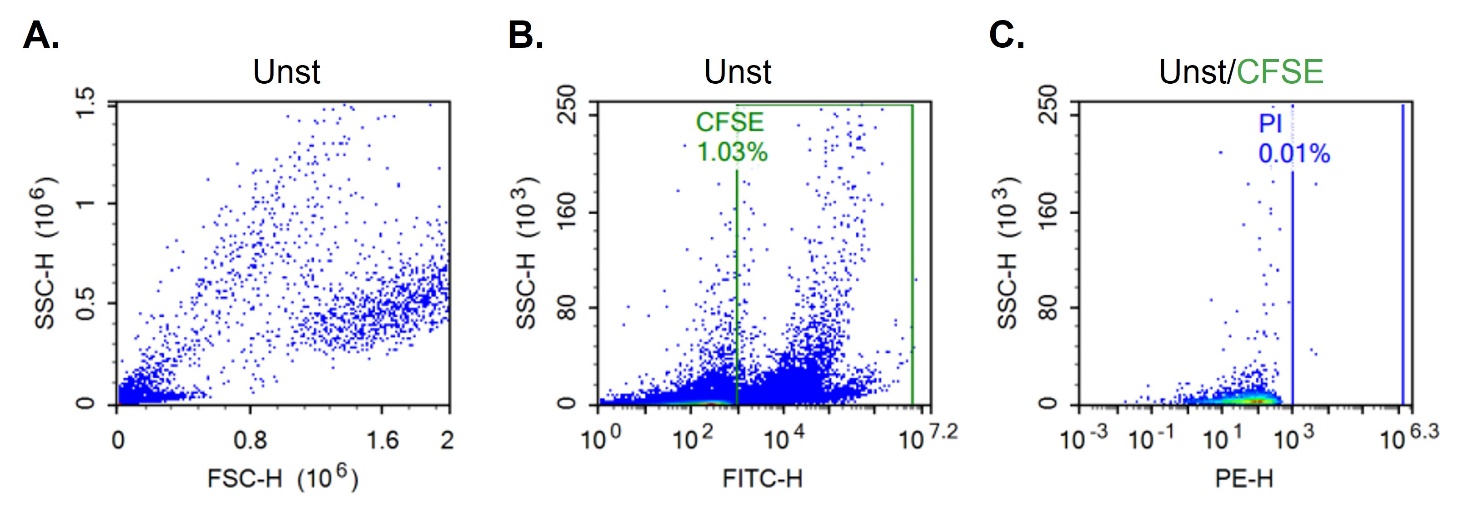
**

**Supplementary Figure 3 – Gating strategy for K562 cells for flow cytometry.** Hierarchical gating to identify cells positive to CFSE (FITC) and PI (PE). a) Representative dot plot diagram of forward-scatter (FSC) and side-scatter (SSC) light. Cells comprise both K562 cells and peripheral blood mononuclear cells. b) Representative dot plot diagram of CFSE^+^ cells. c) Representative dot plot diagram of PI^+^ cells inside CFSE gate (green gate). Plots are for illustration purposes only.

**Supplementary Figure 4.**

**
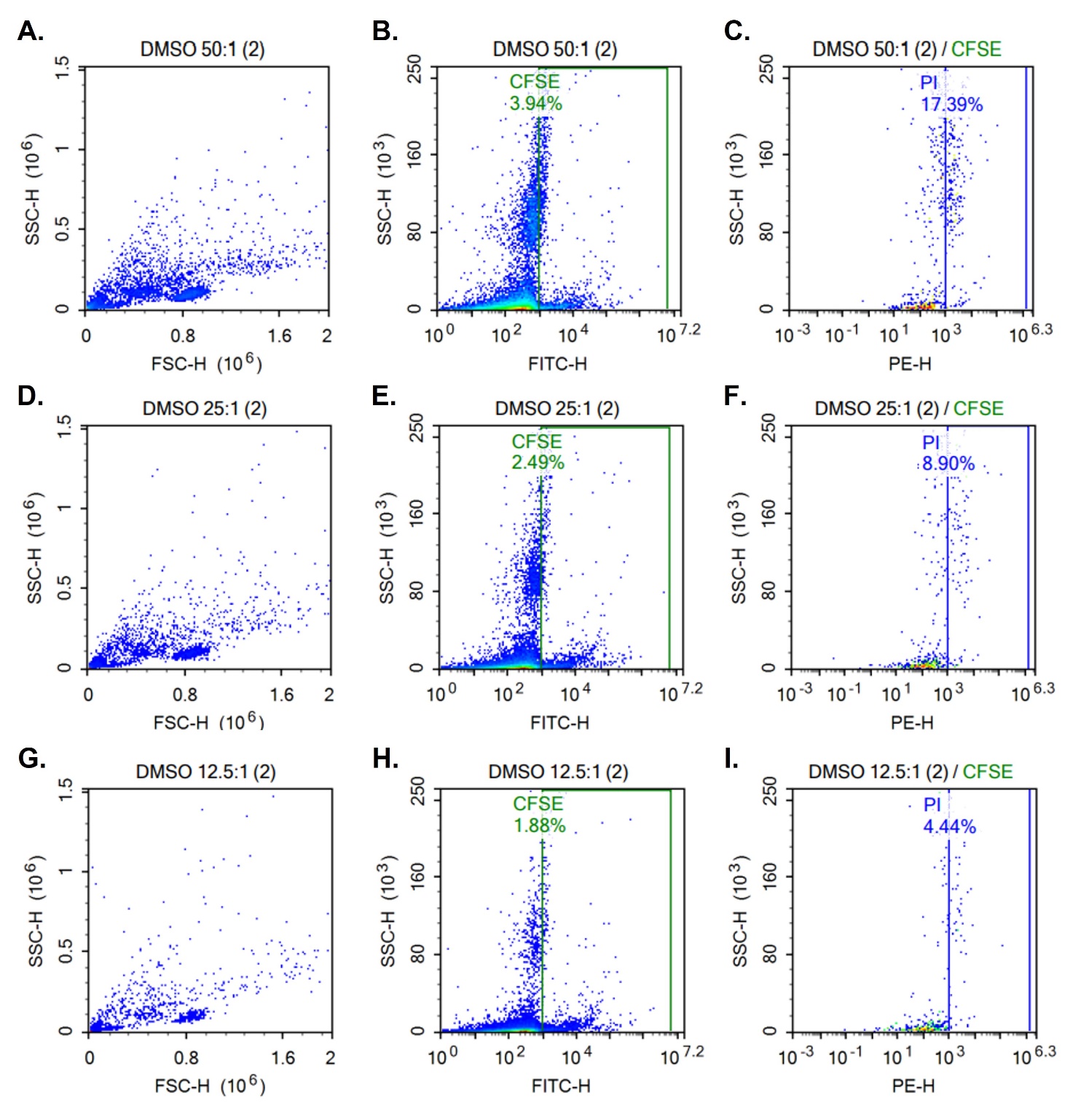
**

**Supplementary Figure 4 – Representative dot plots of DMSO-treated peripheral blood mononuclear cells and cultured with K562 cells.** Representative dot plot diagram of DMSO-treated peripheral blood mononuclear cells cultured with K562 cells at the ratio effector:target of 50:1 (A), 25:1 (D), and 12.5:1 (G). Representative dot plot diagram of CFSE^+^ cells (K562) cultured with DMSO-treated peripheral blood mononuclear cells cultured at the ratio effector:target of 50:1 (B), 25:1 (E), and 12.5:1 (H). Representative dot plot diagram of CFSE^+^PI^+^ cells cultured with DMSO-treated peripheral blood mononuclear cells cultured at the ratio effector:target of 50:1 (C), 25:1 (F), and 12.5:1 (I). Plots are for illustration purposes only.

**Supplementary Figure 5.**


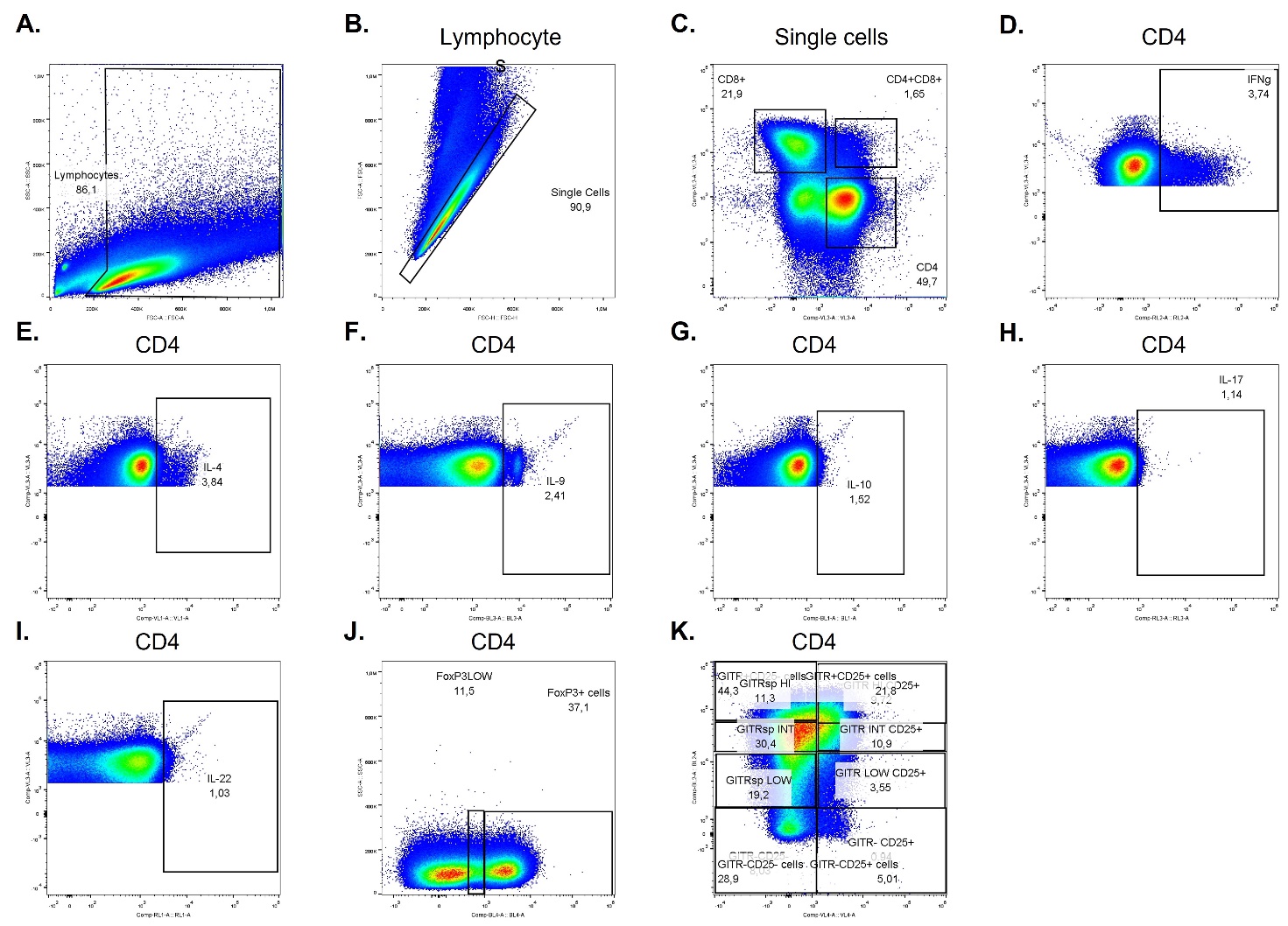


**Supplementary Figure 5 – Gating strategy for lymphocytes for flow cytometry.** Hierarchical gating to identify the different lymphocyte subpopulations. a) Representative dot plot diagram of forward-scatter (FSC) and side-scatter (SSC) light. Lymphocytes gate was created to highlight the main lymphocyte population. b) Representative dot plot diagram of Single cells inside Lymphocytes gate. c) Representative dot plot diagram of CD4^+^, CD8^+^, and CD4^+^CD8^+^ cells inside Single cells gate. Within CD4^+^ and CD8^+^ cells, specific gates to identify cells positive to IFN-γ, IL-4, IL-9, IL-10, IL.17, IL-22, FoxP3, GITR, and CD25 were performed. For simplicity, here only the dot plot diagrams for CD4^+^ cells are shown: CD4^+^IFN-γ^+^ (D), CD4^+^IL-4^+^ (E), CD4^+^IL-9^+^ (F), CD4^+^IL-10^+^ (G), CD4^+^IL-17^+^ (H), CD4^+^IL-22^+^ (I), CD4^+^FoxP3^+^ (J), CD4^+^GITR^+^CD25^+^ (K). j) FoxP3^+^ cells were divided in positive and low, but only FoxP3^+^ cells were shown in graphs. k) GITR^+^CD25^+^ cells comprehend both GITR^high^CD25^+^ and GITR^int^CD25^+^ Plots are for illustration purposes only.

**Supplementary Figure 6.**


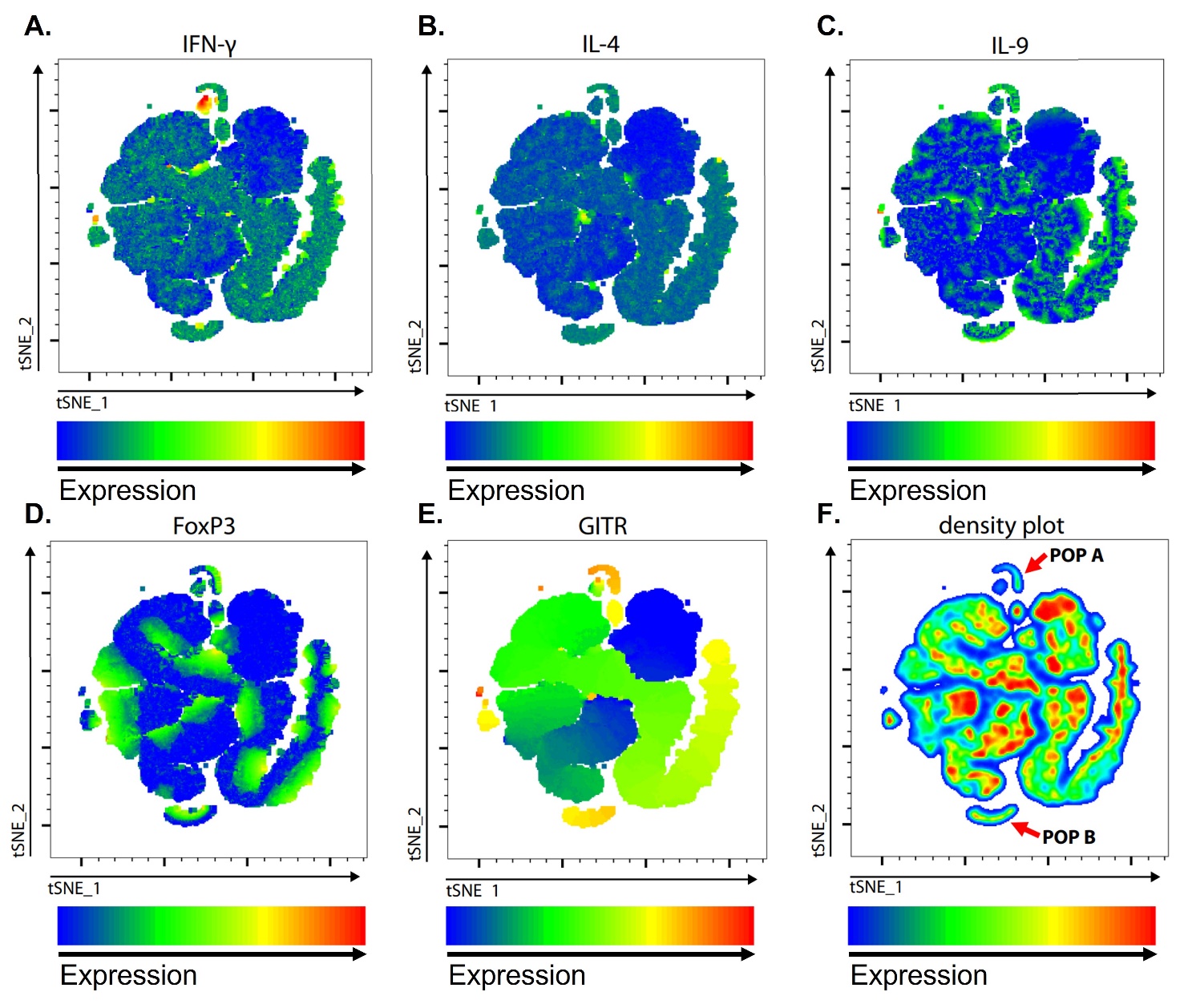


**Supplementary Figure 6 – CD4^+^ cells t-SNE plots for DEP, EE, and PFOS exposure.** Graphical representation of the CD4^+^ cells retrieved through t-SNE analysis following the clustering of flow cytometric data. Merged t-SNE plots of the expression data are shown. The colors range from blue (no expression) to red (maximum expression). a) t-SNE plots of the expression of IFN-γ. b) t-SNE plots of the expression of IL-4. c) t-SNE plots of the expression of IL-9. d) t-SNE plots of the expression of FoxP3. e) t-SNE plots of the expression of GITR. f) density plot of CD4^+^ cells in which the two main populations of interest (POP A and POP B) were highlighted by red arrows.

**Supplementary Figure 7.**


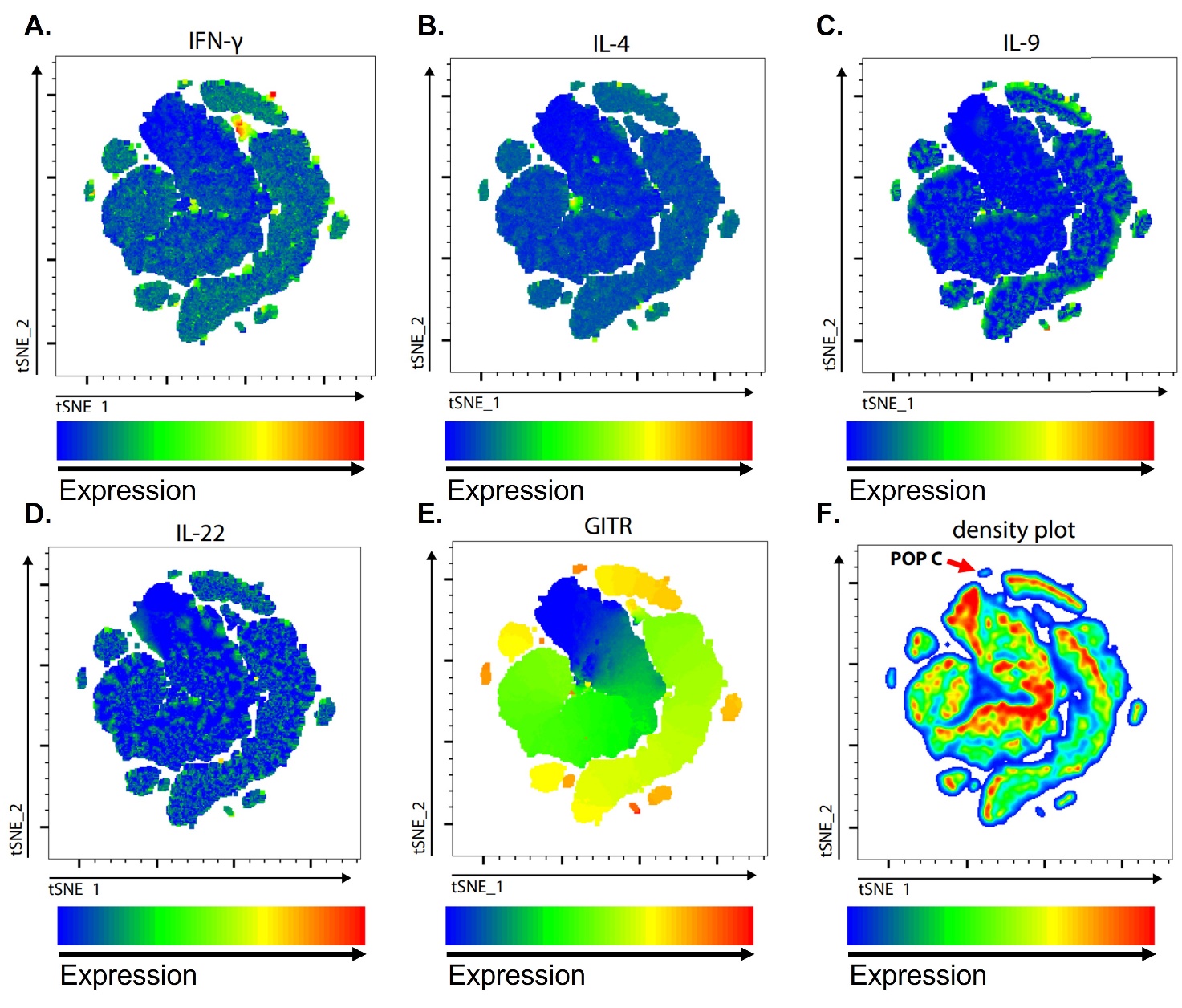


**Supplementary Figure 7 – CD4^+^ cells t-SNE plots for ATR, CYP, and VIN exposure.** Graphical representation of the CD4^+^ cells retrieved through t-SNE analysis following the clustering of flow cytometric data. Merged t-SNE plots of the expression data are shown. The colors range from blue (no expression) to red (maximum expression). a) t-SNE plots of the expression of IFN-γ. b) t-SNE plots of the expression of IL-4. c) t-SNE plots of the expression of IL-9. d) t-SNE plots of the expression of IL-22. e) t-SNE plots of the expression of GITR. f) density plot of CD4^+^ cells in which the main population of interest (POP C) was highlighted by red arrow.

**Supplementary Figure 8.**


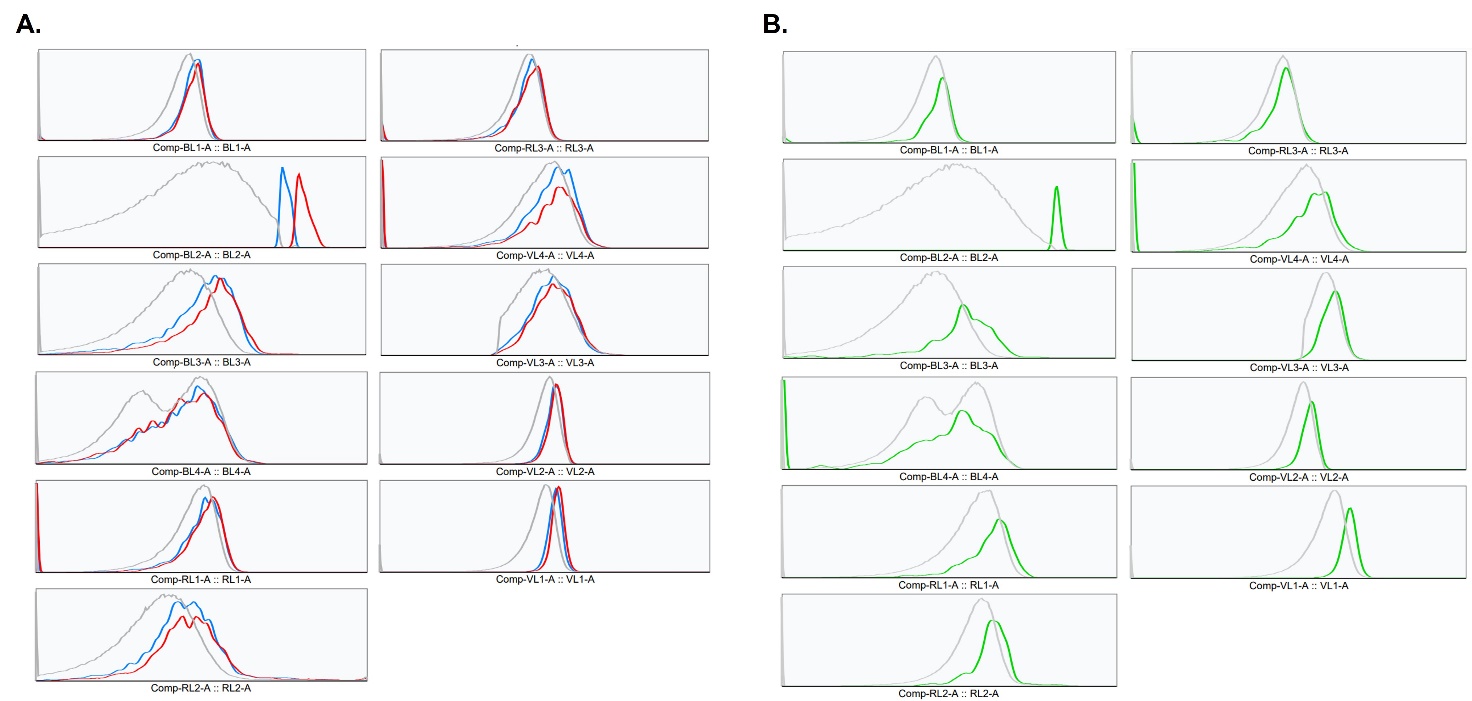


**Supplementary Figure 8 – Phenotype of POP A (red), B (blue), and C (green).** Phenotypic analysis of the 11 markers within CD4^+^ cells retrieved through t-SNE analysis following the clustering of flow cytometric data. a) Phenotypes of POP A and B retrieved following the exposure to DEP, EE, and PFOS. b) Phenotypes of POP C retrieved following the exposure to ATR, CYP, and VIN. Grey line represents other cells. The graphs represent the following markers: BL-1 (IL-10), BL-2 (GITR), BL-3 (IL-9), BL-4 (FoxP3), RL-1 (IL-22), RL-2 (IFN-γ), RL-3 (IL-17A), VL-4 (CD25), VL-3 (CD4), VL-2 (CD8), VL-1 (IL-4).

**Supplementary Table 1**

Values of statistically significantly altered modulation of CD4^+^ subpopulations (log2, expressed as mean ± SD) induced by DEP, EE, and PFOS compared to CTRL. The average abundance (%) of the population (Pop) within CD4^+^ cells is also reported.

|  | | Male donors | | | | Female donors | | | | | |
| --- | --- | --- | --- | --- | --- | --- | --- | --- | --- | --- | --- |
|  |  | Mean ± SD | | % Pop | | | Mean ± SD | | % Pop | | |
| DEP | Pop 7 IFN-γ | | 0.304 ± 0.849 | | 0.33 | | | -0.284 ± 0.091 | | 1.30 |  |
|  | Pop 1 IL-9 | | 0.280 ± 0.846 | | 0.33 | | | -0.292 ± 0.098 | | 1.32 |  |
|  | Pop 3 IL-9 | | 0.090 ± 0.557 | | 0.89 | | | -0.399 ± 0.059 | | 2.76 |  |
|  | Pop 5 IL-9 | | 0.206 ± 0.140 | | 0.24 | | | -0.232 ± 0.054 | | 0.59 |  |
|  | Pop 9 IL-9 | | 0.332 ± 0.089 | | 0.48 | | | -0.151 ± 0.202 | | 0.86 |  |
|  | Pop 10 IL-9 | | 0.410 ± 0.117 | | 0.34 | | | -0.001 ± 0.052 | | 0.50 |  |
|  | Pop 12 IL-9 | | 0.433 ± 0.319 | | 0.35 | | | -0.387 ± 0.199 | | 0.93 |  |
|  | Pop 5 FoxP3 | | 1.095 ± 0.413 | | 0.14 | | | -0.313 ± 0.079 | | 0.48 |  |
|  | Pop 11 FoxP3 | | -1.048 ± 1.835 | | 0.19 | | | -0.514 ± 0.154 | | 0.48 |  |
|  | Pop 14 FoxP3 | | -0.412 ± 0.523 | | 0.21 | | | -0.418 ± 0.123 | | 0.38 |  |
|  | Pop 2 GITR | | 0.312 ± 0.863 | | 0.33 | | | -0.295 ± 0.091 | | 1.31 |  |
|  | Pop 3 GITR | | 0.409 ± 0.329 | | 0.40 | | | -0.340 ± 0.129 | | 1.08 |  |
|  | Pop 5 GITR | | 0.078 ± 0.552 | | 0.90 | | | -0.392 ± 0.064 | | 2.81 |  |
|  | Pop 6 GITR | | 0.053 ± 0.077 | | 15.83 | | | -0.055 ± 0.020 | | 26.74 |  |
|  | Pop 9 GITR | | -0.133 ± 0.045 | | 8.41 | | | 0.106 ± 0.052 | | 6.85 |  |
| EE | Pop 1 IL-4 | | -0.240 ± 0.017 | | 0.24 | | | -0.091 ± 0.382 | | 0.11 |  |
|  | Pop 5 IL-9 | | 0.246 ± 0.146 | | 0.24 | | | -0.208 ± 0.222 | | 0.59 |  |
|  | Pop 7 FoxP3 | | -0.423 ± 0.708 | | 0.69 | | | -0.204 ± 0.064 | | 1.00 |  |
|  | Pop 13 FoxP3 | | 0.195 ± 0.171 | | 0.26 | | | -0.208 ± 0.132 | | 0.40 |  |
| PFOS | Pop 3 IL-9 | | 0.517 ± 0.518 | | 0.89 | | | -0.185 ± 0.021 | | 2.76 |  |
|  | Pop 4 IL-9 | | 0.211 ± 0.415 | | 0.33 | | | -0.244 ± 0.040 | | 0.78 |  |
|  | Pop 9 IL-9 | | 0.410 ± 0.074 | | 0.48 | | | 0.060 ± 0.245 | | 0.86 |  |
|  | Pop 5 FoxP3 | | 1.306 ± 0.738 | | 0.14 | | | -0.302 ± 0.303 | | 0.48 |  |
|  | Pop 11 FoxP3 | | 0.328 ± 0.820 | | 0.19 | | | -0.306 ± 0.073 | | 0.48 |  |
|  | Pop 3 GITR | | 0.373 ± 0.393 | | 0.40 | | | -0.243 ± 0.023 | | 1.08 |  |
|  | Pop 5 GITR | | 0.523 ± 0.520 | | 0.90 | | | -0.172 ± 0.016 | | 2.81 |  |

**Supplementary Table 2**

Values of statistically significantly altered modulation of CD4^+^ subpopulations (log2, expressed as mean ± SD) induced by ATR, CYP, and VIN compared to CTRL. The average abundance (%) of the population (Pop) within CD4^+^ cells is also reported.

|  | | Male donors | | | | Female donors | | | | | |
| --- | --- | --- | --- | --- | --- | --- | --- | --- | --- | --- | --- |
|  |  | Mean ± SD | | % Pop | | | Mean ± SD | | % Pop | | |
| ATR | Pop 6 IFN-γ | | 0.436 ± 1.098 | | 0.08 | | | 0.616 ± 0.205 | | 0.31 |  |
|  | Pop 14 FoxP3 | | -0.153 ± 0.375 | | 0.31 | | | -0.926 ± 0.259 | | 0.39 |  |
| CYP | Pop 5 IFN-γ | | 2.242 ± 0.696 | | 0.08 | | | -0.441 ± 0.763 | | 0.09 |  |
|  | Pop 10 FoxP3 | | 0.532 ± 0.141 | | 0.82 | | | 0.097 ± 0.288 | | 0.97 |  |
|  | Pop 11 GITR | | -0.112 ± 0.070 | | 13.79 | | | 0.059 ± 0.036 | | 10.65 |  |
| VIN | Pop 2 IL-9 | | 0.561 ± 0.380 | | 0.05 | | | 0.656 ± 0.219 | | 0.31 |  |
|  | Pop 2 GITR | | 0.561 ± 0.380 | | 0.05 | | | 0.627 ± 0.136 | | 0.30 |  |
|  | Pop 10 GITR | | -0.050 ± 0.018 | | 16.23 | | | -0.071 ± 0.070 | | 17.78 |  |
|  | Pop 11 GITR | | -0.024 ± 0.026 | | 13.79 | | | 0.099 ± 0.049 | | 10.65 |  |
